# Single-center retrospective study of the effectiveness and toxicity of the oral iron chelating drugs deferiprone and deferasirox

**DOI:** 10.1371/journal.pone.0211942

**Published:** 2019-02-27

**Authors:** Nancy F. Olivieri, Amir Sabouhanian, Brenda L. Gallie

**Affiliations:** 1 Medicine and Public Health Sciences, University of Toronto, Toronto, Ontario, Canada; 2 Toronto General Hospital, University Health Network, Toronto, Ontario, Canada; 3 Department of Ophthalmology and Vision Science, Hospital for Sick Children, Toronto, Ontario, Canada; 4 Departments of Ophthalmology and Vision Science, Medical Biophysics and Molecular Genetics, University of Toronto, Toronto, Ontario, Canada; 5 Techna Institute and Krembil Research Institute, University Health Network, Toronto, Ontario, Canada; Paediatric Centre of Excellence, ZAMBIA

## Abstract

**Background:**

Iron overload, resulting from blood transfusions in patients with chronic anemias, has historically been controlled with regular deferoxamine, but its parenteral requirement encouraged studies of orally-active agents, including deferasirox and deferiprone. Deferasirox, licensed by the US Food and Drug Administration in 2005 based upon the results of randomized controlled trials, is now first-line therapy worldwide. In contrast, early investigator-initiated trials of deferiprone were prematurely terminated after investigators raised safety concerns. The FDA declined market approval of deferiprone; years later, it licensed the drug as “last resort” therapy, to be prescribed only if first-line drugs had failed. We undertook to evaluate the long-term effectiveness and toxicities of deferiprone and deferasirox in one transfusion clinic.

**Methods and findings:**

Under an IRB-approved study, we retrospectively inspected the electronic medical records of consented iron-loaded patients managed between 2009 and 2015 at The University Health Network (UHN), Toronto. We compared changes in liver and heart iron, adverse effects and other outcomes, in patients treated with deferiprone or deferasirox.

**Results:**

Although deferiprone was unlicensed in Canada, one-third (n = 41) of locally-transfused patients had been switched from first-line, licensed therapies (deferoxamine or deferasirox) to regimens of unlicensed deferiprone. The primary endpoint of monitoring in iron overload, hepatic iron concentration (HIC), increased (worsened) during deferiprone monotherapy (mean 10±2–18±2 mg/g; p < 0.0003), exceeding the threshold for life-threatening complications (15 mg iron/g liver) in 50% patients. During deferasirox monotherapy, mean HIC decreased (improved) (11±1–6±1 mg/g; p < 0.0001). Follow-up HICs were significantly different following deferiprone and deferasirox monotherapies (p < 0.0000002). Addition of low-dose deferoxamine (<40 mg/kg/day) to deferiprone did not result in reductions of HIC to <15 mg/g (baseline 20±4 mg/g; follow-up, 18±4 mg/g; p < 0.2) or in reduction in the proportion of patients with HIC exceeding 15 mg/g (p < 0.2). During deferiprone exposure, new diabetes mellitus, a recognized consequence of inadequate iron control, was diagnosed in 17% patients, most of whom had sustained HICs exceeding 15 mg/g for years; one woman died after 13 months of a regimen of deferiprone and low-dose deferasirox. During deferiprone exposure, serum ALT increased over baseline in 65% patients. Mean serum ALT increased 6.6-fold (p < 0.001) often persisting for years. During deferasirox exposure, mean ALT was unchanged (p < 0.84). No significant differences between treatment groups were observed in the proportions of patients estimated to have elevated cardiac iron.

**Conclusions:**

Deferiprone showed ineffectiveness and significant toxicity in most patients. Combination with low doses of first-line therapies did not improve the effectiveness of deferiprone. Exposure to deferiprone, over six years while the drug was unlicensed, in the face of ineffectiveness and serious toxicities, demands review of the standards of local medical practice. The limited scope of regulatory approval of deferiprone, worldwide, should restrict its exposure to the few patients genuinely unable to tolerate the two effective, first-line therapies.

## Introduction

Over 40 years, the iron-chelating agent deferoxamine [1, 2] has transformed previously fatal iron-loading anemias into chronic conditions in high-resource countries [3–7]. The magnitude of the body iron burden is correlated with hepatic iron concentration (HIC) [8]. Thresholds of HIC predict the development of potentially fatal complications [9]: HIC exceeding 15 mg iron/gram liver, dry weight, and sustained elevations of serum ferritin (SF) over 2500 μg/L, increase the risk of premature death [5, 6].

In the 1980s, the parenteral requirement for deferoxamine stimulated studies of orally-active chelators. Deferasirox (Exjade, Novartis) was licensed as first line therapy by the FDA in 2005, following randomized trials comparing this drug, and demonstrating its non-inferiority, to deferoxamine

By contrast, the history of another orally active drug, deferiprone has been characterized by ongoing legal and academic conflict. In 1996, a prolonged controversy arose when a pharmaceutical company, Apotex, terminated two Toronto trials early after the investigators who had initiated both studies raised concerns about effectiveness and safety [10]. This raised issues around the ethics of industry-sponsored clinical trials and institutional conflicts of interest [11, 12]. A randomized controlled trial of deferoxamine and deferiprone, initiated under independent funding and at the direction of the FDA, indicated that deferiprone did not adequately control body iron in many patients [9], but publication of these findings, following repeated legal action by Apotex, did not proceed.

In 2009, 13 years after the two Toronto trials were terminated, the FDA declined Apotex’s request for approval of deferiprone as first-line therapy [13]. Formal inspection of the source data of the Toronto trials had revealed inconsistencies in the data that had been submitted for approval. In 2011, the FDA issued approval for deferiprone as “last resort” therapy, to be prescribed only after both first-line therapies had failed, [14] cautioning that no controlled trials of deferiprone had demonstrated direct treatment benefit. A 2013 Cochrane review asserted that the FDA’s decision “…arose from the lack of new RCT evidence, and the failure to provide answers to the FDA on efficacy and safety.” [15].

The objective of our current study was to compare the effectiveness and toxicity of two iron chelators, deferiprone (Ferriprox; Apotex) and deferasirox (Exjade; Novartis) as used at The University Health Network (UHN) which manages 80% of Canada’s hemoglobinopathy patients [16]. The endpoints were the accepted measurements of iron overload (SF, HIC, and myocardial T2* weighted MRI image, T2*), hepatic and other toxicity, and other patient outcomes, as recorded in the electronic medical record (EMR).

## Methods

### Sample and data collection

In 2000, the UHN Research Ethics Board approved Study REB 01-0170-B, which authorized us to study findings recorded in the EMR of consenting UHN iron-loaded patients. No physical examinations, specimen collections, or other interventions were required. We sought consent from all patients who were regularly transfused at UHN (an estimated 120 patients). Excluded were most patients transfused in other centers and those with sickle cell disease whose transfusion regimens differ from those of patients with thalassemia.

We invited 100 patients to participate in the study; 96 consented. Deferiprone was prescribed to 41 patients with thalassemia major; deferasirox was prescribed to 5 patients with either thalassemia major (52) or Diamond-Blackfan Anemia (4); more than one drug or combination was prescribed to 14 patients (**S1 Data**). This was a retrospective evaluation of the outcomes of iron chelating therapy in clinical practice.

Data up to January 2016 were collected. No data arising from any drug initiated after January 1, 2015 (that is, involving less than one year of therapy) were analyzed.

### Intervals of treatment

During deferiprone exposures, regimens were altered frequently in the same patient over brief periods. Therefore, we sought to avoid erroneous attribution of effectiveness or toxicity to a drug or drug combination, by evaluating treatment intervals. An interval was defined as evaluable if (i) it was bracketed by baseline and follow-up HIC and/or cardiac T2*; and (ii) throughout the interval, one drug or drug combination had been prescribed with no interruption longer than one month. We identified 132 evaluable intervals. Two additional intervals missing follow-up data are included because of serious adverse events (death and agranulocytosis). Sixteen of the 132 intervals (12%) in 12 patients were excluded because of rapid changes in therapy in parallel with absence of assessment of the above endpoints (**S1 Table. Excluded intervals.)**

### Data collected and statistical analysis

Data were recorded from the EMRs according to the protocol, including: indications, if stated, for each regimen; symptoms; adverse effects; baseline and follow-up SF, HIC and T2*; and proportions of intervals in which these measurements varied from established risk thresholds. To compare baseline and follow-up values within each treatment group, paired t-test was used; a two-sample t-test was used between treatment groups. Fisher’s exact test was used to compare proportions of intervals in which values exceeded established risk thresholds. All tests were two-tailed; 0.05 was considered of statistical significance. Minitab Express Statistical Software was used for analyses. To determine the rationale for use of deferiprone in each study patient, we examined the EMR for reference to a research protocol [17], for evidence that the patient had failed standard licensed therapies and evidence of the use of the Special Access Program, Health Canada as a mechanism to provide patients with this unlicensed drug.

## Results

### Treatment intervals

**Fig 1:**
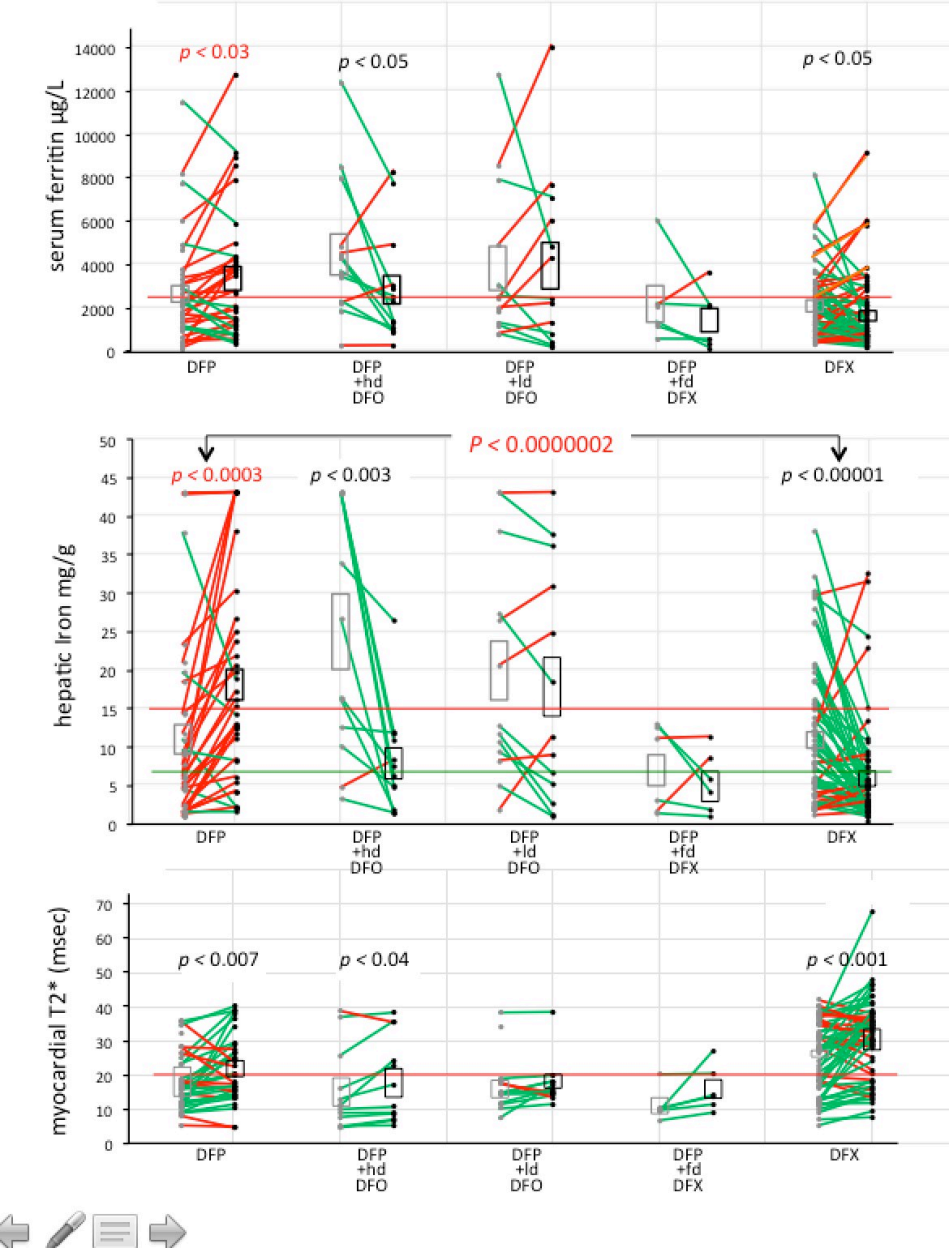
Scatter plots of data points reflecting body iron load. Mean±SEM (range) of values bracketing intervals of deferiprone (DFP) [36] and deferasirox (DFX) [62] monotherapy, and DFP with high dose (hd) [13] and low dose (ld) [13] DFO, and full dose (fd) DFX [6] combination therapies. Grey symbols are baseline (BL) values; black symbols are follow-up (FU) values. BL and FU values for each interval are connected by lines (red lines indicate worsening of iron load over the interval; green lines indicate improvement of iron load over the interval; p values corresponding to worsening body iron are red.

There were 36 monotherapy intervals with deferiprone and 62 with deferasirox. Deferiprone was combined with licensed therapy in another 34 intervals (**S1 Data and Table 1, Fig 1**). Two included treatment intervals are bracketed by SF but not HIC or T2* because of the consequences arising from the exposure to deferiprone (See Results/Adverse Effects/Death, Agranulocytosis). Fourteen patients were analyzable for more than one interval of a drug or drug combination.

**Table 1:** Outcomes of chelation therapies.

Doses of chelator drugs *[recommended dose range*, *mg/kg/d]*		Deferiprone Mono-therapy	Deferiprone with deferoxamine		Deferiprone/ deferasirox	Deferasirox mono-therapy
			Full–dose >40	Low dose <40	Full–dose ≥20	
Deferoxamine *[40–50]*			47±2	24±3		
			(40–55)	(8–37)		
Deferasirox *[20–45]*					29±3	26±1
					(20–42)	(10–45)
Deferiprone *[75–100]*		104±2	101±4	99±5	101±8	
		(75–127)	(76–20)	(75–123)	(69–120)	
Duration (months)		32±4	14±1	21±3	24±4	66±4
SF μg/L	BL	2665±420	4640±904	3823±1021	2204±803	2084±192
		(135–11455)	(220–12373)	(767–12715)	(528–6024)	(339–8117)
	FU	3364±504	2867±718	3949±1113	1465±555	1645±215
		(311–12715)	(232–8267)	(159–14025)	(135–3605)	(161–9127)
	BL vs FU	*p < 0*.*03*	**p < 0.05**	p < 1	p < 0.4	**p < 0.03**
Months between SF		31±4	14±1	22±3	22±5	58±4
Proportion of intervals SF >2500 μg/L	BL	37%	69%	46%	17%	32%
	FU	51%	46%	46%	17%	21%
	BL vs FU	*p <* 0.3	p < 0.5	p < 1	p < 1	p < 0.3
HIC mg/g	BL	10±2(1–43)	25±5(3–4)	20±4(2–43)	7±2(1–13)	11±1(1–38)
	FU	18±2(2–43)	8±2(1–26)	18±4(1–43)	5±2(1–11)	6±1(0.4–33)
	BL vs FU	*p < 0*.*0003*	**p < 0.003**	p < 0.2	p < 0.5	**p < 0.00001**
Months between HIC		30±3	15±2	21±3	26±7	45±3
Proportion of intervals HIC ≤7 mg/g	BL	56%	17%	15%	50%	40%
	FU	24%	50%	38%	67%	76%
	BL vs FU	*p < 0*.*008*	p < 0.1	p < 0.4	p < 1	**p < 0.0001**
Proportion of intervals HIC ≥15 mg/g	BL	21%	67%	46%	0	27%
	FU	50%	8%	46%	0	8%
	*BL vs FU*	*p < 0*.*02*	**p < 0.009**	p < 1	p < 1	**p < 0.02**
Myocardial T2* (msec)	BL	17±4(5–36)	15±4(5–39)	16±2(7–38)	11±2(7–20)	26±1(5–42)
	FU	22±2(5–40)	18±4(5–38)	18±2(12–38)	16±3(9–27)	31±2(8–68)
	*BL vs FU*	p < 0.3	**p < 0.04**	p < 0.2	p < 0.2	**p < 0.001**
Months between T2*		29±4	15±1	15±1	22±6	43±3
Proportion of intervals T2* ≤10 msec	BL	18%	42%	8%	67%	5%
	FU	6%	42%	8%	17%	3%
	*BL vs FU*	p < 0.3	p < 1	p < 1	p < 0.2	p < 0.7
Proportion intervals T2* <20 msec	BL	73%	75%	42%	83%	37%
	FU	50%	58%	42%	66%	23%
	*BL vs FU*	p < 0.1	p < 0.5	p < 1	p < 1	p < 0.2
EF (%)	BL	61±2(48–75)	59±3(37–74)	62±2(53–73)	59±2(54–66)	61±1(48–72)
	FU	60±1(51–73)	61±2(50–67)	64±2(54–75)	61±1(57–66)	61±1(39–69)
	*BL vs FU*	p < 1	p < 0.2	p < 0.2	p < 0.2	p < 1
Months between EF		29±4	15±1	24±3	22±6	43±3

Lab values of evaluable intervals available in S1 Data; mean±SEM (range); serum ferritin, SF; hepatic iron concentration, HIC; cardiac ejection fraction, EF; p value indicating improvement in iron load, bold, green cell; p value indicating worsening iron load, italics, red cell.

We identified 70 intervals of deferiprone, initiated in 2009 or later, in 41 patients: 23 patients had one analyzable interval, 11 had two, 5 had three, and 2 had five. We identified 62 intervals of deferasirox initiated in 2005 or later, in 55 patients; 48 patients had one analyzable interval, 4 had two, and 2 had three. Interval durations varied: HICs during deferiprone treatment combined with full–dose deferoxamine were separated by (mean±SEM) 14±2 months, while during deferasirox monotherapy, intervals were longer (mean±SEM) 45±3 months (**Table 1, Fig 1**) because regimens were switched less frequently.

### Dosing

Deferiprone exceeded the maximum recommended dose [100 mg/kg/day] in 38 (54%) treatment intervals, (mean±SEM and median doses: 113±1, and 114, mg/kg/day). Deferasirox was prescribed at (mean±SEM) 26±1 mg/kg/day [recommended, 20–45 mg/kg/day].

### All patients: Effectiveness

Baseline HIC in the intervals of deferiprone (monotherapy/combinations, n = 70) and of deferasirox (monotherapy, n = 62) were not significantly different (p < 0.1) (**S2 Table. All DFP vs all DFX**). During deferiprone, HIC, SF and the proportion of intervals at or exceeding thresholds of risk did not change (p < 0.1); by contrast, during deferasirox treatment HIC, SF (p <0.0001), and the proportion of intervals exceeding thresholds of risk were significantly reduced (improved) compared to baseline (p < 0.01).

Baseline cardiac T2* was significantly lower in deferiprone- than deferasirox-treatment intervals (p <0.001); a comparable mean change in each group resulted in a persistent difference at follow-up (p < 0.0001). The proportion of intervals in which T2* remained ≤ 10 msec and 20 msec did not change in either group.

### Monotherapies: Deferiprone and deferasirox

Between intervals of deferiprone monotherapy (n = 36) and deferasirox monotherapy (n = 62) (**Table 1**, **Fig 1**, **S1 Data. Lab values of included intervals**), mean baseline HIC and SF were not different (p < 0.7 and p < 0.2 respectively); overall, HIC and SF worsened during deferiprone treatment (p < 0.001 and p < 0.0003, respectively), and improved during deferasirox treatment (p < 0.001 and p < 0.0001, respectively); follow-up HIC values differing between deferiprone and deferasirox monotherapies were highly significant (p < 0.0000002). Similarly, although at baseline the proportion of intervals in which HIC ≥15 mg/g did not differ (p < 0.6), the proportion of intervals more than doubled during deferiprone treatment but declined strikingly during deferasirox treatment and, by follow-up, this represented a 5-fold difference (p < 0.0001). Statistically significant improvements in T2* were observed during both deferiprone and deferasirox monotherapy, but the proportions of intervals in which T2* was ≤10 or ≤20 msec, and the mean ejection fraction, did not change significantly with either treatment (**Table 1**).

There were 20 intervals of deferiprone exposure during which HIC was maintained at/reduced to optimal concentrations (<7 mg/g liver) (**S1 Data**. **Lab values of included intervals**). In 12 of 20 intervals deferiprone was co-prescribed with licensed therapy (in 10, full doses of deferoxamine/deferasirox). In 8 of the 20 deferiprone monotherapy intervals, initial HIC was strikingly low (<2 mg/g) (patients 10, 17, 21, 27, 33); in 4/8 intervals, deferiprone dose was ≥110 mg/kg/day (upper recommended dose 100 mg/kg/day). In 4/8 intervals, follow-up T2* was recorded as improved (after a mean 54 months); the other 4 intervals showed no change or a decline in, or no assessment of T2*. ALT increased in 7/8 intervals (including 6 with initial HIC <2 mg/g) and arthralgias were recorded in 4 intervals. Of the 8 intervals during which optimal HIC was achieved or maintained during deferiprone monotherapy, 4 exceeded 2.5 years, and the others spanned 12 to 26 months. In one interval deferiprone monotherapy reduced HIC >10 mg/g to optimal levels.

Of 36 deferiprone monotherapy intervals (**S1 Data**. **Lab values of evaluable intervals)**, one showed an HIC improvement to the optimal level (patient 4 interval 2, 10.9 to 2.3 mg/g). Two intervals showed a decline in HIC but remained above 7 mg/g **(**patient 18 interval 1, 37.8 to 18.9 mg/g; patient 28 interval 2, 19.6 to 14.2 mg/g). Of 62 deferasirox monotherapy intervals, one showed a poor response with elevation of HIC out of the optimal range (patient 47 interval 1, 5.3 to 13.4 mg/g) and two intervals that were abnormal at baseline worsened (patient 54 interval 1, 12.9 to 22.9 mg/g; patient 80 interval 1, 12.8 to 32.6 mg/g). These iron load changes were similarly reflected in SF.

Because the widespread prescribing of deferiprone had followed a staff change at UHN in 2009, exposure to deferiprone monotherapy (32±4 months) was shorter than to deferasirox monotherapy (66±4 months). To determine if differences were related to longer deferasirox exposures, we also documented relevant endpoints after 30±6 months of deferasirox. The data indicate that the greater effectiveness of deferasirox was unrelated to longer deferasirox exposures (**S3 Table. All patients exposed 30 months)**.

### Deferiprone added to deferoxamine at therapeutic and low doses

Deferiprone was added to therapeutic (≥40 mg/kg/day) deferoxamine (13 intervals), or to low-dose (<40 mg/kg/day) deferoxamine (13 other intervals) (**Table 1, Fig 1**). There was a striking difference between the two groups: after a short period (14.7±1.8 months), the addition of deferoxamine 47.2±1.4 mg/kg/day to deferiprone effected significant improvement in HIC (p < 0.003), T2* (p < 0.034), SF (p < 0.043), and in the proportion of intervals in which HIC exceeded 15 m/g (p < 0.009). By contrast, over 21.1±2.6 months, deferiprone combined with deferoxamine 24.2±2.8 mg/kg/day (equivalent to 4.2 days/week of therapeutic deferoxamine) did not significantly alter HIC, SF, the proportion of intervals in which HIC or SF exceeded risk thresholds, T2*, or the proportions of intervals in which T2* remained ≤10 and ≤20 msec.

### Deferiprone combined with deferasirox at therapeutic and low doses

Similar results emerged during intervals of deferasirox added to deferiprone. When deferasirox 28.9±3.2 mg/kg/day was combined with deferiprone, mean HIC and SF remained within optimal range (Table 1). In six patients a mean increase of approximately 5 msec in T2* did not reach statistical significance (p < 0.1). In only two intervals low dose (<20 mg/kg/day) deferasirox was added to deferiprone; during one interval, the patient (#19) died after 13 months.

### Alterations in transfusion intensity

In seven deferiprone intervals, shortly following introduction of deferiprone, the EMR explicitly documented a plan to reduce volumes of transfused blood to attempt to ameliorate myocardial iron loading.

### Monitoring for agranulocytosis in deferiprone-exposed patients

Agranulocytosis was recognized in early studies to be a risk of deferiprone [18, 19]. Weekly complete blood counts (CBCs) are mandated in the product monograph [20] and the UHN guidelines [21]. In 14 (34%) patient records, the failure to monitor weekly CBCs was noted in the EMR.

### Adverse effects

#### Death

While on deferiprone with low-dose deferasirox over 13 months, patient #19 developed arthralgias, nausea, vomiting, headaches and visual disturbances, and died suddenly, presumably of cardiac failure. Pre-deferiprone HIC was 43 mg/g and T2* was 8.3±0.2 msec; these were not re-assessed over the 13 months of the regimen, in non-compliance with guidelines. Pre-deferiprone SF was 4071 μg/L; this had increased to 8018 μg/L in the months before death, during exposure to a regimen of 100 mg deferiprone/kg/day and alternate-week deferasirox (equivalent to 15 mg/kg/day, <50% of the recommended deferasirox dose for this elevation of HIC).

No death was recorded in deferasirox-treated patients

#### Elevations in serum alanine aminotransferase (ALT)

**Fig 2:**
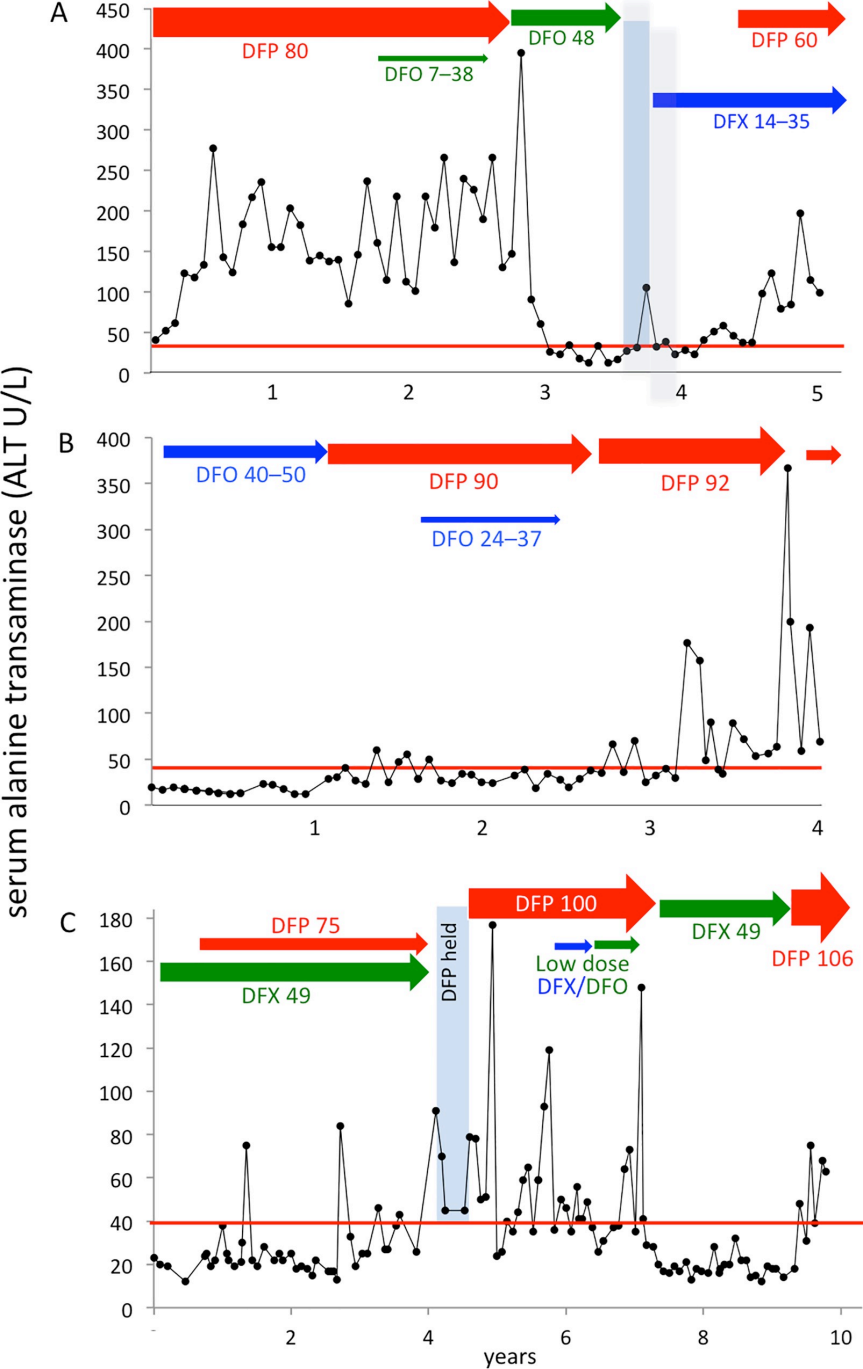
ALT elevations correlating with deferiprone chelation. (A) Patient #34, (B) Patient #1, (C) Patient #19 (datafile). Normal ALT <40U/L, red line; deferiprone (DFP) red arrows and font; deferasirox (DFX), blue arrows and font; deferoxamine (DFO), green arrows and font.

Most ALT elevations above normal (40 units per liter; U/L) observed during deferiprone did not resolve within one interval (or at all); therefore, ALT changes were analyzed in individual patients rather than treatment intervals (**Fig 2, Table 2**).

**Table 2:** Patients with increased ALT (normal <40 U/L) up to January 2016.

Patients [n]	Measurement	Mean±SEM	Median(range)
[26] Increased ALT upon first exposure to DFP [22] or dose escalation [4]; HepC positive [7].	BL ALT	25±4	22(5–75)
	Peak ALT	166±19	139(59–395)
	Fold increase over BL	6.6	6.3(2–24)
	Months between BL and first ALT elevation over BL	3±0.4	2(1–9)
	Months between BL and peak ALT	14.9±2.2	13(1–46)
	FU HIC	15±2	13(1–46)
[21] remained on DFP: [5] ALT normalized; [16] ALT remained >40 U/L.	BL ALT	26±3	24(5–75)
	FU ALT	70±14	55(9–305)
	P value BL to FU	*p < 0*.*01*	
	Fold increase FU over BL	2.7	2.3(1–19)
	FU HIC	15±3	11(2–43)
[16/21] ALT remains abnormal.	BL ALT	25 ± 4	24(9–69)
	FU ALT	87±17	64(41–305)
	P value BL to FU	*p < 0*.*003*	
	Fold increase FU over BL	3.5	2.7(1–19)
	HIC at censure of data	18±4	13(2–43)
	Months on deferiprone	49±6	61(13–75)

DFP, deferiprone; HIC, hepatic liver iron concentration; HepC, hepatitis C virus; Baseline, BL; Follow-up, FU; p value indicating worsening ALT, italics, red cell.

In 40/41 patients evaluable for changes in ALT, three patterns of changes were observed (**Table 2**).

- In 26/40 (65%) evaluable patients, seven positive for hepatitis C, ALT increased over baseline following either introduction of deferiprone (in 22), or dose escalations to 111–127 mg/kg/day (in 4), of deferiprone. Elevations were recorded 3±0.4 months following introduction/escalation; mean peak elevation (6.6-fold; range 2-24-fold) was recorded 14.9±2.2 months following introduction/escalation.
- In 4/40 of evaluable patients, three positive for hepatitis C, elevations of ALT followed deferiprone but fluctuating pre-deferiprone values prevented conclusions about their relationship to deferiprone. One patient died 13 months after introduction of deferiprone with low-dose deferasirox; one resumed deferoxamine monotherapy with a return to baseline ALTs; two remained on deferiprone (one monotherapy; one combined with deferasirox) with a return to baseline ALT values.
- In 10/40 evaluable patients, four positive for hepatitis C, no increases in ALT were observed. Five of the 10 were prescribed full-dose deferoxamine or deferasirox along with deferiprone; five were receiving deferiprone monotherapy.

**Continued deferiprone despite to elevations in ALT.** In 11/26 patients in whom ALTs increased, deferiprone was continued. Eight patients on deferiprone monotherapy had elevated ALT for years. Three patients continued on deferiprone, two combined with deferoxamine at 42 mg/kg/day and 16 mg/kg/day, respectively, and one combined with deferasirox 30 mg/kg/day. ALT elevations returned to baseline in three of the eight after 0.5, 0.5, and 5.5 years of deferiprone.

**Alteration in regimen.** In 15/26 patients in whom ALTs had increased, regimens were altered; in 14/15, ALT elevations resolved completely. In 4/15 patients, deferiprone was withdrawn; ALT returned to pre-deferiprone values within two months. In 3/15, deferiprone dose was reduced (to 70 mg/kg/day, 65 mg/kg/day, and 59 mg/kg/day, respectively); ALT elevations partially (n = 1) or completely (n = 2) resolved over months. In the remaining 8/15 patients, licensed therapy with deferasirox or deferoxamine was added (in parallel, in two patients, with reduction of deferiprone dose); in all 8, ALT returned to pre-deferiprone values within two months.

**Re-challenge.** Ten of 15 patients were re-challenged with their original regimens: two were re-prescribed deferiprone after it had been withdrawn; two re-prescribed full-dose deferiprone after temporary dose reduction; and six re-prescribed deferiprone monotherapy after an added licensed drug was stopped. In all 10, ALT re-surges followed re-challenge; three are shown in **Fig 2**.

Overall, after ALT elevations were observed, deferiprone was continued in 21/26 patients. In five, ALT normalized: during monotherapy (3) or after addition of full-dose deferoxamine (1), or deferasirox (1). In 16 patients at the time of writing, ALT remained abnormal, elevated at 87±17 U/L (3.5-fold over baseline) after 49±6 months of exposure (p < 0.003).

In deferasirox-treated patients, ALT did not change between baseline (33±5 U/L) and follow-up (36±15 U/L; p < 0.84). Seven (12.5%) patients developed elevations over baseline. Four patients continued deferasirox and ALT normalized in three. Three patients resolved on deferoxamine, but rebounded when deferiprone monotherapy was introduced. ALT remained unresolved in two patients but normalized in one patient after 63 months on deferiprone.

#### New diabetes mellitus during deferiprone

Of 36 non-diabetic patients, 6 (16.6%) were diagnosed with new diabetes during deferiprone (as monotherapy or combined with deferoxamine 7–28 mg/kg/day). In 4/6, HICs closest to the diagnosis of diabetes exceeded 20 mg/g. Of 51 deferasirox-exposed, non-diabetic patients, one (2%), whose parents are diabetic, was diagnosed with diabetes after seven years of excellent compliance with deferasirox; HIC closest to the diagnosis of diabetes was 1.6 mg/g.

#### GI disturbances

GI disturbances were reported in 7/41 (17%) of deferiprone-treated patients and 6/55 (11%) of deferasirox-treated patients.

#### Agranulocytosis

Two episodes of agranulocytosis were recorded in patient #3 who 18 years previously developed agranulocytosis requiring hospitalization and infusions of Granulocyte Colony Stimulating Factor (G-CSF) [18] following deferiprone exposure. In 2012, the patient was again prescribed deferiprone monotherapy, despite good ongoing control of iron burden (pre-deferiprone HIC 0.9 mg/g; T2* 16 msec) during deferasirox therapy. Agranulocytosis again required hospitalization and G-CSF. One month after marrow recovery, deferiprone was prescribed a third time; agranulocytosis again required hospitalization and G-CSF. Repeated exposures to deferiprone after agranulocytosis is contraindicated by published guidelines.

#### Neutropenia

Neutopenia (Absolute Neutrophil Count <200) developed in another treatment interval, in a different patient, approximately 3.5 years after the introduction of deferiprone monotherapy. No agranulocytosis or neutropenia were reported in deferasirox-treated patients.

#### Arthralgias

An acknowledged adverse effect associated with deferiprone, arthralgias developed in 11 (27%) patients within months of introduction of deferiprone. This was frequently attributed in the clinical notes to alternative etiologies. In five patients, deferiprone was discontinued: in four patients, 22 to 38 months following onset of arthralgias and, in one patient, after 8 months when HIC was had increased from 23 to 30 mg/g. Arthralgias resolved in all five patients following deferiprone withdrawal. In six patients, deferiprone was not withdrawn; resolution in five was not recorded, and patient #19 complained of arthralgias until death.

#### Elevations in serum creatinine

Serum creatinine values exceeding baseline for longer than one month were recorded in 10 (17.8%) deferasirox-exposed patients; in three, the relationship to deferasirox was uncertain. Five patients were switched to deferiprone; in another four, elevations resolved while deferasirox continued; in the tenth, creatinine peaked at 172 μmol/L, and declined to 130 μmol/L while deferasirox continued. No elevations in serum creatinine were reported during deferiprone.

### Mechanism of prescription of deferiprone

From 2009 to 2015, deferiprone was not licensed in Canada. There are two possible processes by which an unlicensed drug can be used in patient care in Canada: under the terms a formal research protocol of a (registered) clinical trial [17] or under Health Canada’s Special Access Program (SAP) which “considers requests …for access to unauthorized drugs for …serious or life-threatening conditions when conventional therapies … have failed, are unsuitable or unavailable.” [22].

### Indications for prescription of deferiprone

Deferiprone was prescribed to 41 study patients between 2009 and 2015. We could identify in the EMR no explanation for a proposed switch to deferiprone that was supported by evidence of failure of licensed therapy prescribed as recommended. There was no indication that any patient switched to deferiprone over these six years had “failed” therapy with either deferoxamine or deferasirox. Most patients were recorded as tolerant of at least one and (in most), both licensed first-line chelating agents; some had sustained minor adverse events during deferasirox that had resolved by the time deferiprone was prescribed.

Based upon pre-deferiprone HIC and T2*, the 41 study patients switched to deferiprone could be assigned to one of four groups (**S1 Text. Discussing Indications**). Group I patients (16) had demonstrated optimal responses to licensed therapies; Group II patients (3) had a prolonged absence from chelation related to pregnancy, but licensed therapies had not “failed” and were not “unsuitable or unavailable”; Group III patients (13) had inadequate control of HIC concentration (without evidence of cardiac iron loading) in most following treatment with lower-than-recommended doses of licensed therapy; and Group IV patients (9) had reduced cardiac T2*; half the Group IV patients had, in parallel, the highest HIC recorded in the clinic. According to guidelines, this situation is an indication for therapeutic doses of licensed chelation, not deferiprone.

## Discussion

We report the effectiveness and toxicity of the two orally active iron chelator drugs, deferasirox and deferiprone in transfused patients managed at Canada’s largest transfusion program. This was not a prospective trial with study protocols, but rather examination of the EMR of patients who consented.

Between 2009 and 2015, one-third of iron-loaded patient were removed from first-line licensed drugs and prescribed regimens involving unlicensed deferiprone. This represents a higher proportion than expected from the literature on patients who have “failed” licensed therapies [3–5]. We were unable to identify in any patient switched to deferiprone evidence of failure, unsuitability, or unavailability of licensed therapies.

During deferiprone monotherapy, body iron burden increased to levels placing patients at risk for glucose intolerance, cardiac disease, and premature death [5, 6]; one death occurred and new diabetes mellitus, a predictable consequence of uncontrolled iron burden, developed in 17% patients, five times the incidence reported in the modern era of deferoxamine [7]. Body iron burden decreased during deferasirox monotherapy. The frequent elevations in serum ALT observed during exposure to deferiprone in these patients are consistent with our previous observations of hepatic dysfunction arising and hepatic fibrosis progressing during deferiprone therapy, even in patients whose body iron had stabilized [23, 24]. Liver histology was not obtained in the patients in the present study, despite the observed sustained elevations in liver enzymes. Consequently, the etiology and potential hepatic damage associated with the liver enzymes remains undefined [24].

After numerous subsequent studies provided “disparate findings in small series of patients” [15] the FDA approved deferiprone as “last resort” therapy [27], to be prescribed only after all first-line therapies had failed [14], while confirming that no controlled trials had demonstrated a direct treatment benefit [20]. Moreover, the FDA also judged that the year-long study, submitted as ‘pivotal’ evidence for the unique ‘cardio-protective’ effect of deferiprone, [25] did not establish this benefit [26]. Nonetheless, based upon a claimed ‘cardio-protective’ effect [27–29], deferiprone is prescribed worldwide as first line therapy, either as monotherapy (15%), or in regimens combining deferiprone with less-than -therapeutic doses of other drugs (25%) [30, 31]. Deferiprone is frequently prescribed as first line therapy even to children (for example, 68.3% of children in the Middle East [32], despite inadequate monitoring [33–35]). Its recommendation in pediatric practice persists despite higher rates of toxicity in children, including neutropenia (12.6%), agranulocytosis (5%) [35], and liver dysfunction [36, 37]), than were reported in the Apotex-directed “safety” study [34].

The results of this retrospective study of the EMR of patients in one Canadian institution contrast strikingly with the literature. They extend concerns arising from the two prematurely terminated Toronto trials of deferiprone [38], challenging the belief that deferiprone enhances organ-specific iron removal [27–29, 39–41]. In most previous studies of deferiprone, body iron as quantitated by HIC has been unreported, selectively reported, reported after short-term exposures, or reported as unchanged or having worsened [10, 18, 34, 42–74]. By contrast, two Apotex-funded studies have reported declines in HIC during combination therapy (deferiprone combined with deferoxamine) as comparable, or better, than during deferoxamine monotherapy but both studies administered deferoxamine monotherapy at less than 70% therapeutic dose [75, 76]; another uncontrolled study which claimed liver iron declined significantly during combination therapy reported a mean decline (in five patients) which derived from the exceptional reduction of liver iron in one patient.[77].

HIC is the only parameter demonstrated to be directly correlated with body iron [78]. Hence, the lack of HIC data is difficult to reconcile with the idea that body iron is adequately reduced during deferiprone monotherapy or deferiprone when it is combined with less than therapeutic doses of deferoxamine [79].

Rarely discussed with respect to the issue of “combination” therapy is the dose of deferoxamine “combined” with deferiprone. Protocols of deferiprone with low-dose (2–3 infusions/week) deferoxamine, or therapeutic deferoxamine (≥40 mg/kg/day daily) are generally reported under the general rubric of combination therapy, without clarification of dose. For example, deferoxamine doses are reported as “20 to 60 mg/kg/day” [80] or “30 to 45 mg/kg/day, 5 to 7 days per week” [81], which represent between 40% and 120% of recommended dose and therefore cannot be considered a single regimen. No analyses of combination therapy have related the outcomes of therapy to the dose of deferoxamine administered with deferiprone. We were unable to identify a previous publication presenting evidence that deferiprone combined with deferoxamine at a less-than-therapeutic dose (<40 mg/kg/day) adequately reduced body iron burden. Our literature review and our present data by contrast indicate that during combination of deferiprone with deferoxamine, adequate control of body iron is achieved only when full-dose deferoxamine is prescribed. [69, 80–82] This suggests that effectiveness of combination therapy is related to the effectiveness of deferoxamine. Of further concern, combinations of therapy represent off-label prescribing in most jurisdictions, and impose a 3-fold increased risk of adverse events [15].

Despite reports that cardiac iron is ‘uniquely’ improved by deferiprone [25, 27–29, 39, 41, 79, 83], our data comparing deferiprone and deferasirox showed comparable, and modest, changes in cardiac T2*, with no differences in the proportions of patients estimated as having severe cardiac iron loading. Abnormal T2*s persisted, while HICs remained elevated, or increased in elevation, during years of deferiprone exposure. This is entirely consistent with the acknowledged dynamics of slower cardiac iron deposition and removal compared to those of the liver, [84] and confirms that myocardial iron does not decline in isolation if body iron burden (quantitated by liver iron concentration) is insufficiently treated. Importantly, an industry-sponsored study submitted to FDA as “pivotal” to support superior cardio-protection of deferiprone monotherapy [25] failed to provide evidence for this benefit [26]. By contrast, the (only) independently funded, randomized, double-blinded, placebo-controlled study to have been reported in the literature confirmed a lack of superiority in ‘cardio-protection’ of deferiprone when combined with full-dose deferoxamine compared to deferoxamine monotherapy [85].

With respect to T2*, most studies of deferiprone monotherapy, often with extended exposures, report no results, no change, improvements in selected patients only, or non-validated endpoints, and cardiac-related deaths [10, 18, 34, 42–61, 63–65, 69, 73, 74, 86–89]. With respect to combination therapy, most studies have not reported cardiac outcomes [57, 60, 61, 63, 64, 75, 90–103]. Uncontrolled studies often combining deferiprone with full-dose deferoxamine have reported changes, [62, 69, 70, 77, 80, 81, 104–109], but of the eight controlled studies in the literature, seven failed to show any difference in cardiac outcomes between combination therapy and deferoxamine monotherapy [65–67, 72, 73, 82, 85]. The eighth study compared a combination of deferiprone and deferoxamine to deferoxamine monotherapy prescribed at lower than therapeutic doses [76].

We observed another outcome not reported in most studies of deferiprone: new diabetes mellitus occurring during deferiprone exposure, as body iron burdens increased or remained elevated. This dreaded complication of poor iron control [5] developed in nearly 17% of deferiprone-exposed patients. One early (Apotex) study reported an incidence of new diabetes in 3.6% deferiprone-exposed patients [56]; however, the glucose intolerance in more than 50% of patients who prematurely stopped deferiprone in that safety study was not reported.

Hepatic dysfunction as reflected by elevations in serum ALT in 65% of UHN patients, sustained over years (**Table 2**), suggests direct hepato-toxicity. Our original concerns about deferiprone-associated liver toxicity [10] were dismissed [27, 28, 39] [110]; the incidence of deferiprone-associated ALT elevations was subsequently estimated as 7.5% [111]. However, most studies in the literature have failed to report ALT changes, or reported lack of changes after short-term exposures, or in selected patients only [10, 18, 44–47, 49, 51, 52, 56, 58, 62, 66–72, 86, 87, 112–114]. One large study recorded ALT surges 3-fold over baseline in 20% of patients [52]; another reported a 4-fold greater mean ALT elevation in deferiprone-exposed patients, than during deferoxamine, but claimed this to be not significantly different [25]. By contrast, the FDA observed that this observation might “signal[s] the potential for deferiprone induced liver toxicity." Often, “transient” ALT elevations [43, 48, 54, 59] have been later acknowledged as sustained: [51, 59, 60] the Apotex “safety” trial, for example, claimed initially that “increased ALT levels “usually stabilized or regressed after three to six months” [34], but later acknowledged mean ALT had remained significantly elevated over baseline over years [56].

Uniquely, we documented changes in ALT following discontinuation and re-challenge with deferiprone (**Fig 2**). Because liver enzymes reflect hepatocyte integrity rather than liver function [115], and because we did not record albumin or pro-thrombin values, liver functional status cannot be provided. However, our original concerns about deferiprone and hepatocyte integrity are clearly underscored by these findings.

The irregular monitoring for bone marrow toxicity in one-third of this clinic’s patients and the repeated prescribing of deferiprone following episodes of life-threatening agranulocytosis requiring hospitalization violate regulatory agency guidelines, including those of UHN itself [21]. Deaths related to agranulocytosis are reported in under-monitored patients [15] and demand compliance with guidelines specifying with weekly monitoring.

Limitations of this single center analysis include that it does not represent a randomized prospective analysis, a limitation regrettably common in the deferiprone literature. In addition, liver iron concentration and T2* were often not clearly recorded prior to, and following, changes in regimens; as noted, changes were often undertaken without baseline and following data. We circumvented this potential confounder, which would have prevented a clear understanding of the outcomes of effectiveness and safety of different regimens, by defining treatment intervals bracketed by relevant endpoints. Finally, although we have full access to the EMR clinic notes, we are not privy to the reasons why treatment regimens were frequently altered without recording relevant endpoints, or to the rationale behind other clinical decisions not recorded in these notes.

## Conclusion

Between 2009 and 2015, one-third of patients transfused and managed in Canada’s largest transfusion program were switched from first-line, licensed drugs to regimens of unlicensed deferiprone. Although there is suggestion of a research protocol in this clinic in an abstract [116], it appears that Health Canada’s Special Access Program that authorizes use of an unlicensed drug when conventional therapies have failed or are unsuitable or unavailable, was likely used to make deferiprone available to this large proportion of UHN patients. There was no evidence of a failure of first-line therapy in any patient switched to deferiprone.

We provide new evidence of inadequate reduction in hepatic iron, a 17% incidence of new diabetes, and new liver dysfunction in 65% of patients, many who were challenged and re-challenged with deferiprone despite elevated liver enzymes having developed during previous exposure. We identified no evidence of a ‘cardio-protective’ effect during deferiprone therapy.

Resources to examine these concerns about deferiprone in a prospective controlled study, as urged 20 years ago, will never now be made available. In an era when two highly effective, first-line, chelating agents—one orally active—are available, we caution doctors to reserve deferiprone for patients who have genuinely failed other treatments.

Just as important and of concern are questions raised about the procedures by which a large proportion of patients at the largest Research Institute in Canada were switched from licensed therapies to regimens involving unlicensed deferiprone coincidentally corresponding to market approval of deferiprone. The use of Health Canada’s “Special Access Program” and the years of continued exposure of unlicensed deferiprone despite the ineffectiveness and toxicity observed in our study, indicates that issues arising from this analysis are not only scientific. The findings demand an urgent, transparent review of current standards of patient protection, informed consent, and medical practice.

## Supporting information

S1 Table Excluded intervals. (PDF)

S2 Table All DFP vs all DFX. All patients exposed to deferiprone compared to all patients exposed to deferasirox: Lab values indicating iron over-load (mean±SEM); Serum ferritin, SF; Hepatic iron concentration, HIC; (#), number of intervals; baseline, BL; follow-up, FU; significant p values, bold.
(PDF)

S3 Table All patients exposed 30 months. All patients exposed to deferiprone for 31.9±3.7 months compared to all patients exposed to deferasirox at 30±6 months. Lab values of iron over-load (mean±SEM(range)). Serum ferritin, SF; Hepatic iron concentration, HIC; baseline, BL; follow-up, FU; significant p values, bold; p values indicating worsening iron overload, red.
(PDF)

S1 Data Lab values of included intervals. (XLSX)

S1 Text Discussing indications. (PDF)
