# Correction: Single-center retrospective study of the effectiveness and toxicity of the oral iron chelating drugs deferiprone and deferasirox

**DOI:** 10.1371/journal.pone.0214005

**Published:** 2019-03-13

**Authors:** The PLOS ONE Staff

## Notice of republication

An incorrect version of Fig 1 was published in error. The publisher apologizes for this error. This article was republished on March 5, 2019 to correct for this error. Please download this article again to view the correct version. The originally published, uncorrected article and the republished, corrected article are provided here for reference.

## Supporting information

S1 File Originally published, uncorrected article. (PDF)

S2 File Republished, corrected article. (PDF)
