# Supplementary material for: Single-center retrospective study of the effectiveness and toxicity of the oral iron chelating drugs deferiprone and deferasirox
Source: PLoS One. 2019 Feb 27;14(2):e0211942. doi: 10.1371/journal.pone.0211942 (PMC6392256; doi:10.1371/journal.pone.0211942)
Supplement: S1 Table — (PDF) [file pone.0211942.s001.pdf]

S1 Table. All patients exposed to deferiprone compared to all patients exposed to deferasirox (mean±SEM). Serum ferritin, SF; Hepatic iron concentration, HIC; baseline, BL; followu-up, FU

		Deferiprone, All	Deferasirox, All	P value deferiprone vs deferasirox
Number of intervals		70	62	
Number of patients		41	56	
Months exposure		70±2	66±4	
SF µg/L)	BL	3251±348	2084±192	**p < 0.004**
	FU	3282±368	1645±215	**p < 0.001**
		p < 1 vs BL	p < 0.001 vs BL	
Proportion of intervals with SF >2500 µg/L	BL	45%	32%	p < 0.2
	FU	48%	21%	**p < 0.002**
		p < 1 vs BL	p < 0.2 vs BL	
HIC, mg/g dry weight	BL	14(2)	11(1)	p < 0.12
	FU	15(2)	6(1)	**p < 0.001**
		p < 0.1 vs BL	p < 0.0001 vs BL	
Proportion of intervals with HIC >15 mg/g	BL	32%	27%	p < 0.6
	FU	37%	8%	**p < 0.001**
		p < 1 vs BL	p < 0.01 vs BL	
Myocardial T2* (msec)	BL	16(1)	26(1)	**p < 0.001**
	FU	20(1)	31(2)	**p < 0. 001**
		p < 0.001 vs BL	p < 0. 001 vs BL	
Proportion of intervals with T2*≤10 msec	BL	25%	5%	**p < 0.002**
	FU	12%	3%	p < 0.1
		p < 0.1 vs BL	p < 1 vs BL	
Proportion of intervals with T2*<20 msec	BL	78%	37%	**p < 0.001**
	FU	62%	23%	**p < 0.001**
		p < 0.1 vs BL	p < 0.2 vs BL	
