# Supplementary material for: Single-center retrospective study of the effectiveness and toxicity of the oral iron chelating drugs deferiprone and deferasirox
Source: PLoS One. 2019 Feb 27;14(2):e0211942. doi: 10.1371/journal.pone.0211942 (PMC6392256; doi:10.1371/journal.pone.0211942)
Supplement: S2 Table — All patients exposed to deferiprone compared to all patients exposed to deferasirox: Lab values indicating iron over-load (mean±SEM); Serum ferritin, SF; Hepatic iron concentration, HIC; (#), number of intervals; baseline, BL; follow-up, FU; significant p values, bold. (PDF) [file pone.0211942.s002.pdf]

S2 Table. All patients exposed to deferiprone compared to all patients exposed to deferasirox: Lab values indicating iron over-load (mean±SEM); Serum ferritin, SF; Hepatic iron concentration, HIC; (#), number of intervals; baseline, BL; follow-up, FU; significant p values, **bold.**

		Deferiprone All	Deferasirox All	
Number of intervals		70	62	p value deferiprone vs deferasirox
Number of patients		41	56	
Months exposure		70±2	66±4	
SF(µg/L)	BL	3251±348	2084±192	**p < 0.004**
	FU	3282±368	1645±215	**p < 0.001**
	BL vs FU	p < 1 vs BL	**p < 0.001** vs BL	
Proportion of intervals with SF >2500 µg/L	FU	45%	32%	p < 0.2
	BL	48%	21%	**p < 0.002**
	BL vs FU	p < 1 vs BL	p < 0.2 vs BL	
HIC mg/g dry weight	BL	14(2)	11(1)	p < 0.12
	FU	15(2)	6(1)	**p < 0.001**
	BL vs FU	p < 0.1	**p < 0.0001**	
Proportion of intervals with HIC >15 mg/g	BL	32%	27%	p < 0.6
	FU	37%	8%	**p < 0.001**
	BL vs FU	p < 1	**p < 0.01**	
Myocardial T2* (msec)	BL	16(1)	26(1)	**p < 0.001**
	FU	20(1)	31(2)	**p < 0. 001**
	BL vs FU	**p < 0.001**	**p < 0. 001**	
Proportion of intervals with T2*≤10 msec	BL	25%	5%	**p < 0.002**
	FU	12%	3%	p < 0.1
	BL vs FU	p < 0.1	p < 1	
Proportion of intervals with T2*<20 msec	BL	78%	37%	**p < 0.001**
	FU	62%	23%	**p < 0.001**
	BL vs FU	p < 0.1	p < 0.2	
