# Supplementary material for: Single-center retrospective study of the effectiveness and toxicity of the oral iron chelating drugs deferiprone and deferasirox
Source: PLoS One. 2019 Feb 27;14(2):e0211942. doi: 10.1371/journal.pone.0211942 (PMC6392256; doi:10.1371/journal.pone.0211942)
Supplement: S3 Table — All patients exposed to deferiprone for 31.9±3.7 months compared to all patients exposed to deferasirox at 30±6 months. Lab values of iron over-load (mean±SEM(range)). Serum ferritin, SF; Hepatic iron concentration, HIC; baseline, BL; follow-up, FU; significant p values, bold; p values indicating worsening iron overload, red. (PDF) [file pone.0211942.s003.pdf]

S3 Table. All patients exposed 30 months. All patients exposed to deferiprone for 31.9±3.7 months compared to all patients exposed to deferasirox at 30±6 months. Lab values of iron over-load (mean±SEM(range)). Serum ferritin, SF; Hepatic iron concentration, HIC; baseline, BL; follow-up, FU; significant p values, bold; p values indicating worsening

		Deferiprone	Deferasirox	p value deferiprone vs deferasirox
Deferasirox dose (mg/kg/day)		–	25.3±0.9(10–38)	
Deferiprone dose (mg/kg/day)		103.9±2.3(75–127)	–	
Age at start (years)		33.8±1.6(21.3–60.2)	29.6±1.3(13.4– 59)	
Duration of therapy (months)		31.9±3.7	*See individual parameters*	
SF(µg/L)	BL	2665±420(135–11455)	2113±202(339–8117)	$p < 0.24$
	FU	3364 ± 504(311– 12715)	1600±162(159–6102)	**$p < 0.002$**
	BL vs FU	***$p < 0.0238$***	***$p < 0.037$***	
Proportion of intervals with SF >2500 µg/L	FU	37%	33%	$p < 0.38$
	BL	51%	21%	**$p < 0.0001$**
	BL vs FU	*$p < 0.34$*	*$p < 0.22$*	
Months between SF		30.6±3.7	33.6±0.7	
HIC mg/g dry weight	BL	10.5±1.9(0.9–43)	10.4±1.1(1.1–38.1)	$p < 0.96$
	FU	18.2±2.3(1.5–43)	6.3±0.9(0.9–32.8)	**$p < 0.0001$**
	BL vs FU	***$p < 0.0003$***	***$p < 0.0005$***	
Proportion of intervals HIC ≤7 mg/g	BL	56%	44%	$p < 0.28$
	FU	24%	74%	**$p < 0.0001$**
	BL vs FU	***$p < 0.0045$***	***$p < 0.002$***	
Proportion of intervals with HIC >15 mg/g	BL	20.5%	22%	$p < 1.0$
	FU	50%	9%	***$p < 0.0001$***
	BL vs FU	***$p < 0.002$***	*$p < 0.1$*	
Months between HIC		29.9±3.2	31.8±0.9	
Myocardial T2* (msec)	BL	17.4±1.4(5.3–35.9)	25.9±1.4(10.9–41.9)	**$p < 0.0001$**
	FU	21.5±1.7(4.6–40.0)	29.9±1.3(11.8–48)	**$p < 0.0006$**
	BL vs FU	***$p < 0.03$***	***$p < 0.03$***	
Proportion of intervals with T2*≤10 msec	BL	17.60%	No patients	N/A
	FU	6%	No patients	N/A
	BL vs FU	*$p < 0.26$*		
Proportion of intervals with T2*<20 msec	BL	73%	37%	**$p < 0.002$**
	FU	50%	23%	**$p < 0.012$**
	BL vs FU	*$p < 0.08$*	*$p < 0.16$*	
Months between myocardial T2*s		28.5±3.7	32.6±1.4	
