# Supplementary material for: Single-center retrospective study of the effectiveness and toxicity of the oral iron chelating drugs deferiprone and deferasirox
Source: PLoS One. 2019 Feb 27;14(2):e0211942. doi: 10.1371/journal.pone.0211942 (PMC6392256; doi:10.1371/journal.pone.0211942)
Supplement: S1 Text — (PDF) [file pone.0211942.s005.pdf]

S1 Text. Discussing Indications

Further insight into the important issue of the putative indications for deferiprone has been is limited by the inability to secure information as to under what mechanism deferiprone, an unlicensed drug, was prescribed for six years to over 40% of regularly-reviewed thalassemia patients at UHN. In Canada, access to an unlicensed drug through the regulator (Health Canada) "Special Access Program" (SAP) is generally restricted to provision of drug for five to 10 patients. The SAP permits access *"to non-marketed drugs .... when conventional therapies have failed, are unsuitable, or unavailable ...[thereby authorizing] ... a manufacturer to sell a drug that cannot otherwise be sold or distributed in Canada"*. Access to larger numbers may, by contrast to the situation under the US FDA, be permitted by Health Canada. We originally believed that the primary reason to switch patients to deferiprone from licensed therapy was that the company wanted to gain market approval in Canada and that patients were switched to deferiprone under the SAP. However, the UHN has recently affirmed that the mechanism under which deferiprone was prescribed over these six years was that of a *clinical research study.* One difficulty in this claim is that any evidence of such a study being registered cannot be located. (*In Canada, all clinical trials that are part of a new drug application, i.e., an application to have a new drug approved in Canada must be registered. Trials are registered by the sponsor: an individual, corporate body, institution or organization that conducts a clinical trial. This includes individuals, companies, institutions or organizations that take responsibility for the initiation, management and/or financing of a clinical trial. Individual physicians can be identified as being the sponsor).*

Were the patients treated with deferiprone via SAP because they had developed toxicity to "failed" or were "unsuitable or unavailable"? According to EMR records, each patient was tolerant of at least one and (in most patients), *both* licensed first-line chelating agents.

Elevations in serum creatinine were recorded in 10 deferasirox-exposed patients. All of these patients had been successfully treated with deferoxamine and could have restarted this licensed, first-line drug. Five were switched to deferiprone. In three of the five patients, elevations in creatinine were subsequently documented related to be related to: (i) self-supplementation of oral creatine; (ii) urinary tract infection; and (iii) renal carcinoma, and not likely due to deferasirox. In the other two patients, deferiprone resulted in 3- and 7-fold increases in liver iron to dangerous concentrations but deferiprone was continued for extended periods.

Three other patients developed ***ALT elevations*** during deferasirox. After elevations in ALT were observed during deferasirox therapy, one patient restarted deferoxamine (following the recommendation of a different physician). However in 2009 this patient (with maintained normal ALTs for years) was switched to deferiprone monotherapy. Elevations in ALT persisted while deferiprone was continued. In two other patients,

ALT had remained normal during long-term deferasirox, until unexplained increases in deferasirox doses to those exceeding recommended doses were followed by increases in serum ALT. Both patients were tolerant to deferoxamine but were introduced to and continued on deferiprone monotherapy.

Four patients reported GI symptoms during deferasirox therapy, resumed deferoxamine with good control of body iron and then, without further symptoms, were switched to deferiprone. Deferiprone was continued in one patient while GI symptoms continued. The other patient with GI complaints during deferasirox therapy was switched to deferiprone monotherapy; GI complaints persisted. After biliary disease was diagnosed the patient underwent cholecystectomy, but deferiprone was continued. The third patient had tolerated deferasirox for 22 months developed GI upset after increases in deferasirox doses exceeding those recommended, despite optimal control of body iron; the patient was not treated with first line deferoxamine, to which he was tolerant and compliant for 30 years, but with last-resort deferiprone. The fourth patient with abdominal discomfort during deferasirox and (although also tolerant to deferoxamine) was switched to deferiprone, experienced unfortunate results: liver iron persisted over optimal concentrations and new diabetes was diagnosed after 44 months of deferiprone monotherapy; it is not clear in the EMR why deferoxamine had not been introduced during this prolonged period of poor iron control.

Skin rash was observed following introduction of deferasirox in two patients; this trivial complaint was followed by an immediate switch to deferiprone monotherapy, but in both, liver iron concentration increased unacceptably and both patients resumed deferasirox, without further dermatologic complaints.

Were patients introduced to deferiprone because of poor iron control? To examine the possibility that poor responses to other chelators prompted a switch to deferiprone in any patient, we recorded the liver iron concentration and T2* prior to introduction of deferiprone. Based upon these endpoints each patient could be assigned to one of four groups.

Group I (n=16): pre-deferiprone HIC and T2* consistent with excellent control of body iron Pre-deferiprone liver iron concentration was 2.9 ± 0.4 mg/g (values of HIC and T2* are expressed in mean ±SEM). Pre-deferiprone T2* was 17.4 ± 1.9 msec. All T2*s exceeded 10 msec. Critically, prior to deferiprone in 12 (75%) of these 16 patients, T2* had increased during licensed therapies - by 5.8 ± 1.9 msec over 21.5 ± 4 months. In another 3 (19%) of these 16 patients, pre-deferiprone T2* had remained stable (at 20, 18, and 11.8 msec, respectively). In the 16th patient, liver iron concentration was optimal, but pre-deferiprone T2* had declined (22 to 16 msec) during a year of *low-dose* deferasirox. Deferoxamine (which had controlled body iron for 15 years and with which the patient was compliant) was not re-prescribed.

Group 2 (n= 3): Post pregnancy patients In three patients, pre-deferiprone liver iron concentrations were substantially elevated [39.4 ± 4.5 mg/g], while T2* was acceptable [31.7 ± 3.8 msec]. In none of these three patients had deferasirox and deferoxamine "failed" or was "unsuitable or unavailable". All were iron loaded precisely *because* (due to pregnancy) all iron chelation therapy had been *deliberate*ly withheld for safety reasons for 9-12 months.

In **Group 3 (n=13): Pre-deferiprone HICs were elevated [18.5 ± 3.4 mg/g]; T2*s acceptable** [20.2 ± 2.2 msec]; Prior to deferiprone in four of 13 patients [31%] liver iron concentration had declined substantially [*from 24.5±3.1 to 12.4±1.5 mg/g* over 12.2± 2.9 months]; T2* had increased or was stable, during deferoxamine or deferasirox. In 9 of the 13 patients (69%) pre-deferiprone compliance with licensed chelation was recorded as varying from "poor" to "excellent"; in one patient, deferasirox compliance was recorded as "perfect". Many of these patients were, however, for unknown reasons, prescribed doses of deferoxamine/deferasirox (by the current physician) below those recommended for these levels of body iron burden: deferoxamine was prescribed at 34±4 mg/kg/day and deferasirox at 26.3±1.9 mg/kg/day. (One patient was treated with both low-dose deferasirox and deferoxamine). Overall, pre-deferiprone, responses to these doses of licensed drugs were modest: increases in T2* were recorded in three patients (increases of 1 msec, 5.7 msec, and 7 msec, over 11 months, 23 months, and 7 months, respectively); T2* remained acceptable [at 22±3.4 msec] in another six patients; pre-deferiprone T2* was not recorded in another three patients. In the 13th patient, following a decline in T2* during pregnancy, deferasirox had induced a rapid decline in HIC and a slower increase in T2* at the time deferiprone was introduced.

In **Group 4 (n=9), T2* was reduced; liver iron concentration variable**: In 4 of these 9 patients, pre-deferiprone liver iron concentration was optimal [3.6 ± 0.8 mg/g]. In another 4 of the 9 patients, all pre-deferiprone liver iron concentrations exceeded 40 mg/g, among the highest ever recorded in the Program. (Liver iron in the 9th patient, 12.5 mg/g, was obtained six months *after* deferiprone was introduced). T2* was ≤10 msec in 8 of the 9. In the 9th patient, T2* (<10 msec) was determined only six months after deferiprone was introduced. Pre-deferiprone, 4 of these 8 patients had experienced *improvements* in T2* (1.3±0.2 msec over 11.7±3.2 months) during licensed therapies. Pre-deferiprone, in another 3 of the 8 patients, T2* was *unchanged* during licensed therapy (after six months, 12 months, and 12 months, following a previous assessment). In the last patient, T2* had declined during one year of deferiprone co-administered deferasirox (equivalent to 15 mg/kg/day, about 50% of recommended dose); the patient died on this combination of therapies, as outlined under **Deaths**.
