# Supplementary material for: Correction: Single-center retrospective study of the effectiveness and toxicity of the oral iron chelating drugs deferiprone and deferasirox
Source: PLoS One. 2019 Mar 13;14(3):e0214005. doi: 10.1371/journal.pone.0214005 (PMC6415843; doi:10.1371/journal.pone.0214005)
Supplement: S1 File — (PDF) [file pone.0214005.s001.pdf]

PLOS ONE

RESEARCH ARTICLE

Single-center retrospective study of the effectiveness and toxicity of the oral iron chelating drugs deferiprone and deferasirox

Nancy F. Olivieri[1,2]*, **Amir Sabouhanian**[1,2], **Brenda L. Gallie**[3,4,5]

1 Medicine and Public Health Sciences, University of Toronto, Toronto, Ontario, Canada, **2** Toronto General Hospital, University Health Network, Toronto, Ontario, Canada, **3** Department of Ophthalmology and Vision Science, Hospital for Sick Children, Toronto, Ontario, Canada, **4** Departments of Ophthalmology and Vision Science, Medical Biophysics and Molecular Genetics, University of Toronto, Toronto, Ontario, Canada, **5** Techna Institute and Krembil Research Institute, University Health Network, Toronto, Ontario, Canada

* nancy@hemoglobal.org

Abstract

Background

Iron overload, resulting from blood transfusions in patients with chronic anemias, has historically been controlled with regular deferoxamine, but its parenteral requirement encouraged studies of orally-active agents, including deferasirox and deferiprone. Deferasirox, licensed by the US Food and Drug Administration in 2005 based upon the results of randomized controlled trials, is now first-line therapy worldwide. In contrast, early investigator-initiated trials of deferiprone were prematurely terminated after investigators raised safety concerns. The FDA declined market approval of deferiprone; years later, it licensed the drug as "last resort" therapy, to be prescribed only if first-line drugs had failed. We undertook to evaluate the long-term effectiveness and toxicities of deferiprone and deferasirox in one transfusion clinic.

Methods and findings

Under an IRB-approved study, we retrospectively inspected the electronic medical records of consented iron-loaded patients managed between 2009 and 2015 at The University Health Network (UHN), Toronto. We compared changes in liver and heart iron, adverse effects and other outcomes, in patients treated with deferiprone or deferasirox.

Results

Although deferiprone was unlicensed in Canada, one-third (n = 41) of locally-transfused patients had been switched from first-line, licensed therapies (deferoxamine or deferasirox) to regimens of unlicensed deferiprone. The primary endpoint of monitoring in iron overload, hepatic iron concentration (HIC), increased (worsened) during deferiprone monotherapy (mean 10±2–18±2 mg/g; $p < 0.0003$), exceeding the threshold for life-threatening complications (15 mg iron/g liver) in 50% patients. During deferasirox monotherapy, mean HIC decreased (improved) (11±1–6±1 mg/g; $p < 0.0001$). Follow-up HICs were significantly

OPEN ACCESS

Citation: Olivieri NF, Sabouhanian A, Gallie BL (2019) Single-center retrospective study of the effectiveness and toxicity of the oral iron chelating drugs deferiprone and deferasirox. PLoS ONE 14 (2): e0211942. https://doi.org/10.1371/journal.pone.0211942

Editor: Godfrey Biemba, Paediatric Centre of Excellence, ZAMBIA

Received: July 14, 2018

Accepted: January 25, 2019

Published: February 27, 2019

Copyright: © 2019 Olivieri et al. This is an open access article distributed under the terms of the Creative Commons Attribution License, which permits unrestricted use, distribution, and reproduction in any medium, provided the original author and source are credited.

Data Availability Statement: All relevant data are within the paper and its Supporting Information files.

Funding: The authors received no specific funding for this work.

Competing interests: Dr. Nancy F. Olivieri led two investigator-initiated trials of deferiprone begun in 1989 (partially funded by Apotex Inc. from 1993) at Toronto's Hospital for Sick Children and the University of Toronto. In 1995, she identified concerns regarding long-term effectiveness and

safety of deferiprone. In 1996, both Toronto trials were terminated abruptly and prematurely, and Dr. Olivieri was threatened with legal remedies should she make concerns public. Dr. Brenda L. Gallie, an independent researcher at The Hospital for Sick Children and the University, was involved directly in all related events over the subsequent decade of legal proceedings. An independent description of the first five years of this controversy provides additional details (Thompson J, Baird P, Downie J. The Olivieri Report: The complete text of the report of the independent committee of inquiry commissioned by the Canadian Association of University Teachers. Toronto: James Lorimer & Co. Publishers; 2001). In 2014, a final settlement respecting all legal matters was mediated between Dr. Olivieri and Apotex. This does not alter the authors' adherence to all PLOS ONE policies on sharing data and materials.

different following deferiprone and deferasirox monotherapies ($p < 0.0000002$). Addition of low-dose deferoxamine (<40 mg/kg/day) to deferiprone did not result in reductions of HIC to <15 mg/g (baseline 20±4 mg/g; follow-up, 18±4 mg/g; $p < 0.2$) or in reduction in the proportion of patients with HIC exceeding 15 mg/g ($p < 0.2$). During deferiprone exposure, new diabetes mellitus, a recognized consequence of inadequate iron control, was diagnosed in 17% patients, most of whom had sustained HICs exceeding 15 mg/g for years; one woman died after 13 months of a regimen of deferiprone and low-dose deferasirox. During deferiprone exposure, serum ALT increased over baseline in 65% patients. Mean serum ALT increased 6.6-fold ($p < 0.001$) often persisting for years. During deferasirox exposure, mean ALT was unchanged ($p < 0.84$). No significant differences between treatment groups were observed in the proportions of patients estimated to have elevated cardiac iron.

Conclusions

Deferiprone showed ineffectiveness and significant toxicity in most patients. Combination with low doses of first-line therapies did not improve the effectiveness of deferiprone. Exposure to deferiprone, over six years while the drug was unlicensed, in the face of ineffectiveness and serious toxicities, demands review of the standards of local medical practice. The limited scope of regulatory approval of deferiprone, worldwide, should restrict its exposure to the few patients genuinely unable to tolerate the two effective, first-line therapies.

Introduction

Over 40 years, the iron-chelating agent deferoxamine [1, 2] has transformed previously fatal iron-loading anemias into chronic conditions in high-resource countries [3–7]. The magnitude of the body iron burden is correlated with hepatic iron concentration (HIC) [8]. Thresholds of HIC predict the development of potentially fatal complications [9]: HIC exceeding 15 mg iron/gram liver, dry weight, and sustained elevations of serum ferritin (SF) over 2500 μg/L, increase the risk of premature death [5, 6].

In the 1980s, the parenteral requirement for deferoxamine stimulated studies of orally-active chelators. Deferasirox (Exjade, Novartis) was licensed as first line therapy by the FDA in 2005, following randomized trials comparing this drug, and demonstrating its non-inferiority, to deferoxamine

By contrast, the history of another orally active drug, deferiprone has been characterized by ongoing legal and academic conflict. In 1996, a prolonged controversy arose when a pharmaceutical company, Apotex, terminated two Toronto trials early after the investigators who had initiated both studies raised concerns about effectiveness and safety [10]. This raised issues around the ethics of industry-sponsored clinical trials and institutional conflicts of interest [11, 12]. A randomized controlled trial of deferoxamine and deferiprone, initiated under independent funding and at the direction of the FDA, indicated that deferiprone did not adequately control body iron in many patients [9], but publication of these findings, following repeated legal action by Apotex, did not proceed.

In 2009, 13 years after the two Toronto trials were terminated, the FDA declined Apotex's request for approval of deferiprone as first-line therapy [13]. Formal inspection of the source data of the Toronto trials had revealed inconsistencies in the data that had been submitted for approval. In 2011, the FDA issued approval for deferiprone as "last resort" therapy, to be

prescribed only after both first-line therapies had failed, [14] cautioning that no controlled trials of deferiprone had demonstrated direct treatment benefit. A 2013 Cochrane review asserted that the FDA's decision ". . .arose from the lack of new RCT evidence, and the failure to provide answers to the FDA on efficacy and safety." [15].

The objective of our current study was to compare the effectiveness and toxicity of two iron chelators, deferiprone (Ferriprox; Apotex) and deferasirox (Exjade; Novartis) as used at The University Health Network (UHN) which manages 80% of Canada's hemoglobinopathy patients [16]. The endpoints were the accepted measurements of iron overload (SF, HIC, and myocardial T2* weighted MRI image, T2*), hepatic and other toxicity, and other patient outcomes, as recorded in the electronic medical record (EMR).

Methods

Sample and data collection

In 2000, the UHN Research Ethics Board approved Study REB 01-0170-B, which authorized us to study findings recorded in the EMR of consenting UHN iron-loaded patients. No physical examinations, specimen collections, or other interventions were required. We sought consent from all patients who were regularly transfused at UHN (an estimated 120 patients). Excluded were most patients transfused in other centers and those with sickle cell disease whose transfusion regimens differ from those of patients with thalassemia.

We invited 100 patients to participate in the study; 96 consented. Deferiprone was prescribed to 41 patients with thalassemia major; deferasirox was prescribed to 5 patients with either thalassemia major (52) or Diamond-Blackfan Anemia (4); more than one drug or combination was prescribed to 14 patients (**S1 Data**). This was a retrospective evaluation of the outcomes of iron chelating therapy in clinical practice.

Data up to January 2016 were collected. No data arising from any drug initiated after January 1, 2015 (that is, involving less than one year of therapy) were analyzed.

Intervals of treatment

During deferiprone exposures, regimens were altered frequently in the same patient over brief periods. Therefore, we sought to avoid erroneous attribution of effectiveness or toxicity to a drug or drug combination, by evaluating treatment intervals. An interval was defined as evaluable if (i) it was bracketed by baseline and follow-up HIC and/or cardiac T2*; and (ii) throughout the interval, one drug or drug combination had been prescribed with no interruption longer than one month. We identified 132 evaluable intervals. Two additional intervals missing follow-up data are included because of serious adverse events (death and agranulocytosis). Sixteen of the 132 intervals (12%) in 12 patients were excluded because of rapid changes in therapy in parallel with absence of assessment of the above endpoints (**S1 Table. Excluded intervals.)**

Data collected and statistical analysis

Data were recorded from the EMRs according to the protocol, including: indications, if stated, for each regimen; symptoms; adverse effects; baseline and follow-up SF, HIC and T2*; and proportions of intervals in which these measurements varied from established risk thresholds. To compare baseline and follow-up values within each treatment group, paired t-test was used; a two-sample t-test was used between treatment groups. Fisher's exact test was used to compare proportions of intervals in which values exceeded established risk thresholds. All tests were two-tailed; 0.05 was considered of statistical significance. Minitab Express Statistical

Software was used for analyses. To determine the rationale for use of deferiprone in each study patient, we examined the EMR for reference to a research protocol [17], for evidence that the patient had failed standard licensed therapies and evidence of the use of the Special Access Program, Health Canada as a mechanism to provide patients with this unlicensed drug.

Results

Treatment intervals

There were 36 monotherapy intervals with deferiprone and 62 with deferasirox. Deferiprone was combined with licensed therapy in another 34 intervals (**S1 Data and Table 1, Fig 1**). Two included treatment intervals are bracketed by SF but not HIC or T2* because of the consequences arising from the exposure to deferiprone (See Results/Adverse Effects/Death, Agranulocytosis). Fourteen patients were analyzable for more than one interval of a drug or drug combination.

We identified 70 intervals of deferiprone, initiated in 2009 or later, in 41 patients: 23 patients had one analyzable interval, 11 had two, 5 had three, and 2 had five. We identified 62 intervals of deferasirox initiated in 2005 or later, in 55 patients; 48 patients had one analyzable interval, 4 had two, and 2 had three. Interval durations varied: HICs during deferiprone treatment combined with full–dose deferoxamine were separated by (mean±SEM) 14±2 months, while during deferasirox monotherapy, intervals were longer (mean±SEM) 45±3 months (**Table 1**, **Fig 1**) because regimens were switched less frequently.

Dosing

Deferiprone exceeded the maximum recommended dose [100 mg/kg/day] in 38 (54%) treatment intervals, (mean±SEM and median doses: 113±1, and 114, mg/kg/day). Deferasirox was prescribed at (mean±SEM) 26±1 mg/kg/day [recommended, 20–45 mg/kg/day].

All patients: Effectiveness

Baseline HIC in the intervals of deferiprone (monotherapy/combinations, n = 70) and of deferasirox (monotherapy, n = 62) were not significantly different ($p < 0.1$) (**S2 Table. All DFP vs all DFX**). During deferiprone, HIC, SF and the proportion of intervals at or exceeding thresholds of risk did not change ($p < 0.1$); by contrast, during deferasirox treatment HIC, SF ($p < 0.0001$), and the proportion of intervals exceeding thresholds of risk were significantly reduced (improved) compared to baseline ($p < 0.01$).

Baseline cardiac T2* was significantly lower in deferiprone- than deferasirox-treatment intervals ($p < 0.001$); a comparable mean change in each group resulted in a persistent difference at follow-up ($p < 0.0001$). The proportion of intervals in which T2* remained $\leq$ 10 msec and 20 msec did not change in either group.

Monotherapies: Deferiprone and deferasirox

Between intervals of deferiprone monotherapy (n = 36) and deferasirox monotherapy (n = 62) (**Table 1**, **Fig 1**, **S1 Data. Lab values of included intervals**), mean baseline HIC and SF were not different ($p < 0.7$ and $p < 0.2$ respectively); overall, HIC and SF worsened during deferiprone treatment ($p < 0.001$ and $p < 0.0003$, respectively), and improved during deferasirox treatment ($p < 0.001$ and $p < 0.0001$, respectively); follow-up HIC values differing between deferiprone and deferasirox monotherapies were highly significant ($p < 0.0000002$). Similarly, although at baseline the proportion of intervals in which HIC $\geq$15 mg/g did not differ ($p < 0.6$), the proportion of intervals more than doubled during deferiprone treatment but

Table 1. Outcomes of chelation therapies.

**Doses of chelator drugs *[recommended dose range, mg/kg/d]***		**Deferiprone Mono-therapy**	**Deferiprone with deferoxamine**		**Deferiprone/ deferasirox**	**Deferasirox mono-therapy**
			**Full-dose >40**	**Low dose <40**	**Full-dose ≥20**	
Deferoxamine *[40–50]*			47±2	24±3		
			(40–55)	(8–37)		
Deferasirox *[20–45]*					29±3	26±1
					(20–42)	(10–45)
Deferiprone *[75–100]*		104±2	101±4	99±5	101±8	
		(75–127)	(76–20)	(75–123)	(69–120)	
Duration (months)		32±4	14±1	21±3	24±4	66±4
SF μg/L	BL	2665±420	4640±904	3823±1021	2204±803	2084±192
		(135–11455)	(220–12373)	(767–12715)	(528–6024)	(339–8117)
	FU	3364±504	2867±718	3949±1113	1465±555	1645±215
		(311–12715)	(232–8267)	(159–14025)	(135–3605)	(161–9127)
	BL vs FU	*$p < 0.03$*	**$p < 0.05$**	$p < 1$	$p < 0.4$	**$p < 0.03$**
Months between SF		31±4	14±1	22±3	22±5	58±4
Proportion of intervals SF >2500 μg/L	BL	37%	69%	46%	17%	32%
	FU	51%	46%	46%	17%	21%
	BL vs FU	$p < 0.3$	$p < 0.5$	$p < 1$	$p < 1$	$p < 0.3$
HIC mg/g	BL	10±2(1–43)	25±5(3–4)	20±4(2–43)	7±2(1–13)	11±1(1–38)
	FU	18±2(2–43)	8±2(1–26)	18±4(1–43)	5±2(1–11)	6±1(0.4–33)
	BL vs FU	*$p < 0.0003$*	**$p < 0.003$**	$p < 0.2$	$p < 0.5$	**$p < 0.00001$**
Months between HIC		30±3	15±2	21±3	26±7	45±3
Proportion of intervals HIC ≤7 mg/g	BL	56%	17%	15%	50%	40%
	FU	24%	50%	38%	67%	76%
	BL vs FU	*$p < 0.008$*	$p < 0.1$	$p < 0.4$	$p < 1$	**$p < 0.0001$**
Proportion of intervals HIC ≥15 mg/g	BL	21%	67%	46%	0	27%
	FU	50%	8%	46%	0	8%
	BL vs FU	*$p < 0.02$*	**$p < 0.009$**	$p < 1$	$p < 1$	**$p < 0.02$**
Myocardial T2* (msec)	BL	17±4(5–36)	15±4(5–39)	16±2(7–38)	11±2(7–20)	26±1(5–42)
	FU	22±2(5–40)	18±4(5–38)	18±2(12–38)	16±3(9–27)	31±2(8–68)
	BL vs FU	$p < 0.3$	**$p < 0.04$**	$p < 0.2$	$p < 0.2$	**$p < 0.001$**
Months between T2*		29±4	15±1	15±1	22±6	43±3
Proportion of intervals T2* ≤10 msec	BL	18%	42%	8%	67%	5%
	FU	6%	42%	8%	17%	3%
	BL vs FU	$p < 0.3$	$p < 1$	$p < 1$	$p < 0.2$	$p < 0.7$
Proportion intervals T2* <20 msec	BL	73%	75%	42%	83%	37%
	FU	50%	58%	42%	66%	23%
	BL vs FU	$p < 0.1$	$p < 0.5$	$p < 1$	$p < 1$	$p < 0.2$
EF (%)	BL	61±2(48–75)	59±3(37–74)	62±2(53–73)	59±2(54–66)	61±1(48–72)
	FU	60±1(51–73)	61±2(50–67)	64±2(54–75)	61±1(57–66)	61±1(39–69)
	BL vs FU	$p < 1$	$p < 0.2$	$p < 0.2$	$p < 0.2$	$p < 1$

(*Continued*)

Table 1. (Continued)

**Doses of chelator drugs *[recommended dose range, mg/kg/d]***		**Deferiprone Mono-therapy**	**Deferiprone with deferoxamine**		**Deferiprone/ deferasirox**	**Deferasirox mono-therapy**
			**Full–dose >40**	**Low dose <40**	**Full–dose ≥20**	
Months between EF		29±4	15±1	24±3	22±6	43±3

Lab values of evaluable intervals available in S1 Data; mean±SEM (range); serum ferritin, SF; hepatic iron concentration, HIC; cardiac ejection fraction, EF; p value indicating improvement in iron load, bold, green cell; p value indicating worsening iron load, italics, red cell.

https://doi.org/10.1371/journal.pone.0211942.t001

declined strikingly during deferasirox treatment and, by follow-up, this represented a 5-fold difference ($p < 0.0001$). Statistically significant improvements in T2* were observed during both deferiprone and deferasirox monotherapy, but the proportions of intervals in which T2* was ≤10 or ≤20 msec, and the mean ejection fraction, did not change significantly with either treatment (**Table 1**).

There were 20 intervals of deferiprone exposure during which HIC was maintained at/ reduced to optimal concentrations (<7 mg/g liver) (**S1 Data**. **Lab values of included intervals**). In 12 of 20 intervals deferiprone was co-prescribed with licensed therapy (in 10, full doses of deferoxamine/deferasirox). In 8 of the 20 deferiprone monotherapy intervals, initial HIC was strikingly low (<2 mg/g) (patients 10, 17, 21, 27, 33); in 4/8 intervals, deferiprone dose was ≥110 mg/kg/day (upper recommended dose 100 mg/kg/day). In 4/8 intervals, follow-up T2* was recorded as improved (after a mean 54 months); the other 4 intervals showed no change or a decline in, or no assessment of T2*. ALT increased in 7/8 intervals (including 6 with initial HIC <2 mg/g) and arthralgias were recorded in 4 intervals. Of the 8 intervals during which optimal HIC was achieved or maintained during deferiprone monotherapy, 4 exceeded 2.5 years, and the others spanned 12 to 26 months. In one interval deferiprone monotherapy reduced HIC >10 mg/g to optimal levels.

Of 36 deferiprone monotherapy intervals (**S1 Data**. **Lab values of evaluable intervals**), one showed an HIC improvement to the optimal level (patient 4 interval 2, 10.9 to 2.3 mg/g). Two intervals showed a decline in HIC but remained above 7 mg/g (patient 18 interval 1, 37.8 to 18.9 mg/g; patient 28 interval 2, 19.6 to 14.2 mg/g). Of 62 deferasirox monotherapy intervals, one showed a poor response with elevation of HIC out of the optimal range (patient 47 interval 1, 5.3 to 13.4 mg/g) and two intervals that were abnormal at baseline worsened (patient 54 interval 1, 12.9 to 22.9 mg/g; patient 80 interval 1, 12.8 to 32.6 mg/g). These iron load changes were similarly reflected in SF.

Because the widespread prescribing of deferiprone had followed a staff change at UHN in 2009, exposure to deferiprone monotherapy (32±4 months) was shorter than to deferasirox monotherapy (66±4 months). To determine if differences were related to longer deferasirox exposures, we also documented relevant endpoints after 30±6 months of deferasirox. The data indicate that the greater effectiveness of deferasirox was unrelated to longer deferasirox exposures (**S3 Table**. **All patients exposed 30 months**).

Deferiprone added to deferoxamine at therapeutic and low doses

Deferiprone was added to therapeutic (≥40 mg/kg/day) deferoxamine (13 intervals), or to low-dose (<40 mg/kg/day) deferoxamine (13 other intervals) (**Table 1**, **Fig 1**). There was a striking difference between the two groups: after a short period (14.7±1.8 months), the addition of deferoxamine 47.2±1.4 mg/kg/day to deferiprone effected significant improvement in HIC ($p < 0.003$), T2* ($p < 0.034$), SF ($p < 0.043$), and in the proportion of intervals in which

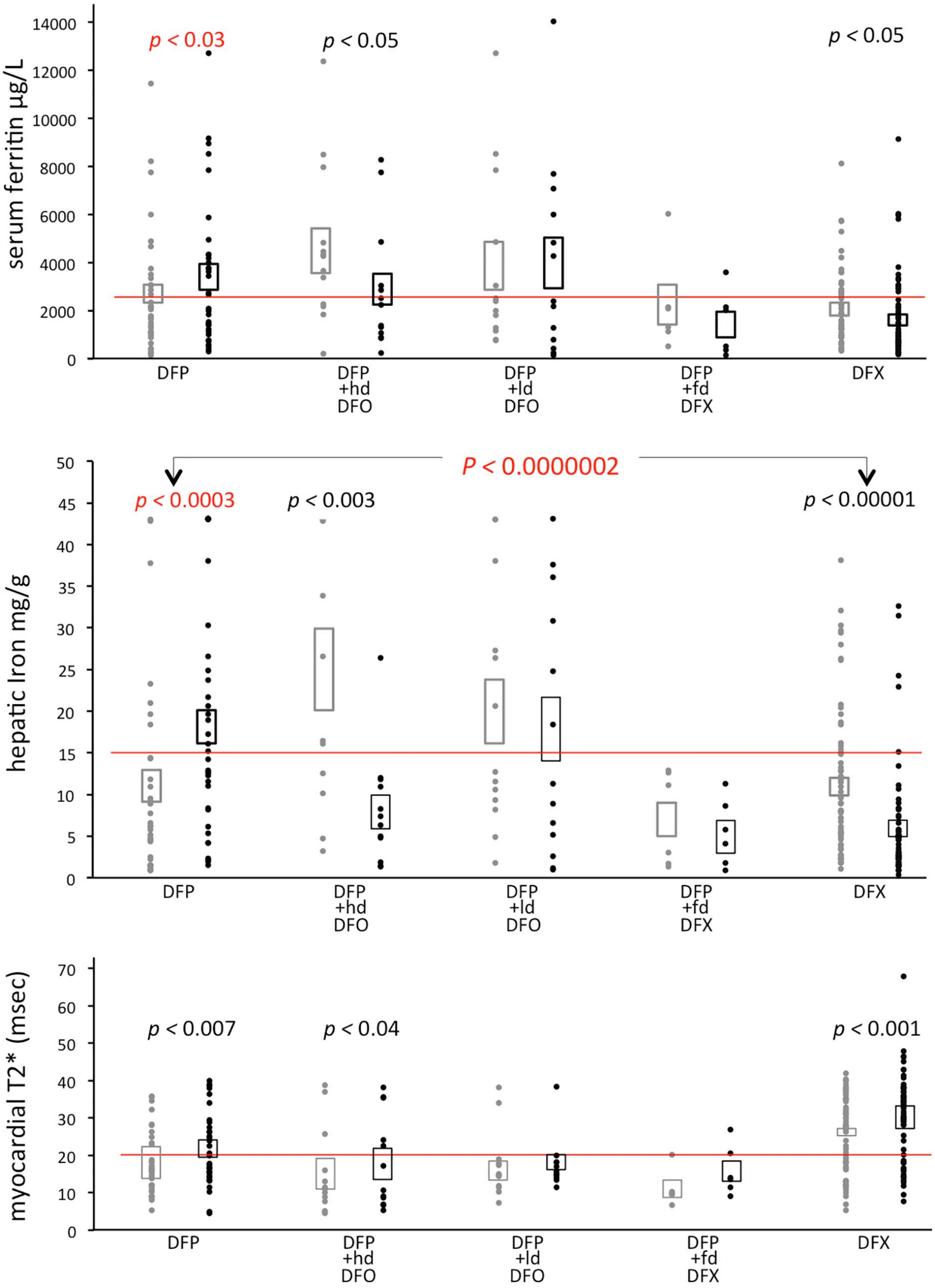

serum ferritin µg/L
14000
12000
10000
8000
6000
4000
2000
0
p < 0.03
p < 0.05
p < 0.05
DFP
DFP +hd DFO
DFP +ld DFO
DFP +fd DFX
DFX
hepatic Iron mg/g
50
45
40
35
30
25
20
15
10
5
0
P < 0.0000002
p < 0.0003
p < 0.003
p < 0.00001
DFP
DFP +hd DFO
DFP +ld DFO
DFP +fd DFX
DFX
myocardial T2* (msec)
70
60
50
40
30
20
10
0
p < 0.007
p < 0.04
p < 0.001
DFP
DFP +hd DFO
DFP +ld DFO
DFP +fd DFX
DFX

Fig 1. Scatter plots of data points reflecting body iron load. Mean±SEM (range) of values bracketing intervals of deferiprone (DFP) [36] and deferasirox (DFX) [62] monotherapy, and DFP with high dose (hd) [13] and low dose (ld) [13] DFO, and full dose (fd) DFX [6] combination therapies. Grey symbols are baseline (BL) values; black symbols are follow-up (FU) values. BL and FU values for each interval are connected by lines (red lines indicate worsening of iron load over the interval; green lines indicate improvement of iron load over the interval; p values corresponding to worsening body iron are red.

https://doi.org/10.1371/journal.pone.0211942.g001

HIC exceeded 15 m/g ($p < 0.009$). By contrast, over 21.1±2.6 months, deferiprone combined with deferoxamine 24.2±2.8 mg/kg/day (equivalent to 4.2 days/week of therapeutic deferoxamine) did not significantly alter HIC, SF, the proportion of intervals in which HIC or SF exceeded risk thresholds, T2*, or the proportions of intervals in which T2* remained ≤10 and ≤20 msec.

Deferiprone combined with deferasirox at therapeutic and low doses

Similar results emerged during intervals of deferasirox added to deferiprone. When deferasirox 28.9±3.2 mg/kg/day was combined with deferiprone, mean HIC and SF remained within optimal range (Table 1). In six patients a mean increase of approximately 5 msec in T2* did not reach statistical significance ($p < 0.1$). In only two intervals low dose (<20 mg/kg/day) deferasirox was added to deferiprone; during one interval, the patient (#19) died after 13 months.

Alterations in transfusion intensity

In seven deferiprone intervals, shortly following introduction of deferiprone, the EMR explicitly documented a plan to reduce volumes of transfused blood to attempt to ameliorate myocardial iron loading.

Monitoring for agranulocytosis in deferiprone-exposed patients

Agranulocytosis was recognized in early studies to be a risk of deferiprone [18, 19]. Weekly complete blood counts (CBCs) are mandated in the product monograph [20] and the UHN guidelines [21]. In 14 (34%) patient records, the failure to monitor weekly CBCs was noted in the EMR.

Adverse effects

Death. While on deferiprone with low-dose deferasirox over 13 months, patient #19 developed arthralgias, nausea, vomiting, headaches and visual disturbances, and died suddenly, presumably of cardiac failure. Pre-deferiprone HIC was 43 mg/g and T2* was 8.3±0.2 msec; these were not re-assessed over the 13 months of the regimen, in non-compliance with guidelines. Pre-deferiprone SF was 4071 μg/L; this had increased to 8018 μg/L in the months before death, during exposure to a regimen of 100 mg deferiprone/kg/day and alternate-week deferasirox (equivalent to 15 mg/kg/day, <50% of the recommended deferasirox dose for this elevation of HIC).

No death was recorded in deferasirox-treated patients

Elevations in serum alanine aminotransferase (ALT). Most ALT elevations above normal (40 units per liter; U/L) observed during deferiprone did not resolve within one interval (or at all); therefore, ALT changes were analyzed in individual patients rather than treatment intervals (**Fig 2**, **Table 2**).

In 40/41 patients evaluable for changes in ALT, three patterns of changes were observed (**Table 2**).

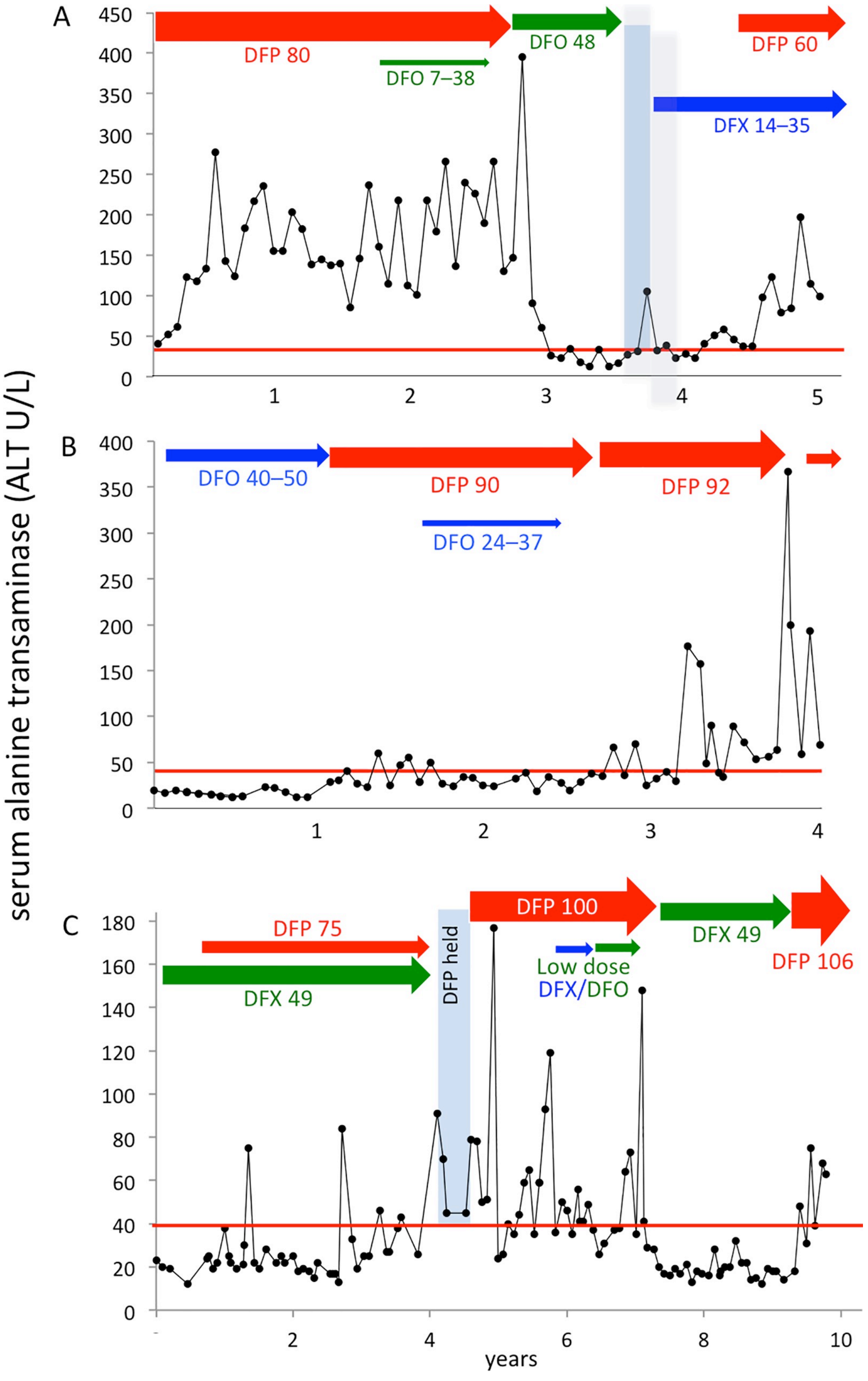
A
DFP 80
DFO 7–38
DFO 48
DFP 60
DFX 14–35
B
DFO 40–50
DFP 90
DFO 24–37
DFP 92
C
DFP 75
DFX 49
DFP held
DFP 100
Low dose
DFX/DFO
DFX 49
DFP 106
serum alanine transaminase (ALT U/L)
years

Fig 2. ALT elevations correlating with deferiprone chelation. (A) Patient #34, (B) Patient #1, (C) Patient #19 (datafile). Normal ALT <40U/L, red line; deferiprone (DFP) red arrows and font; deferasirox (DFX), blue arrows and font; deferoxamine (DFO), green arrows and font.

https://doi.org/10.1371/journal.pone.0211942.g002

i. In 26/40 (65%) evaluable patients, seven positive for hepatitis C, ALT increased over baseline following either introduction of deferiprone (in 22), or dose escalations to 111–127 mg/kg/day (in 4), of deferiprone. Elevations were recorded 3±0.4 months following introduction/escalation; mean peak elevation (6.6-fold; range 2-24-fold) was recorded 14.9±2.2 months following introduction/escalation.

ii. In 4/40 of evaluable patients, three positive for hepatitis C, elevations of ALT followed deferiprone but fluctuating pre-deferiprone values prevented conclusions about their relationship to deferiprone. One patient died 13 months after introduction of deferiprone with low-dose deferasirox; one resumed deferoxamine monotherapy with a return to baseline ALTs; two remained on deferiprone (one monotherapy; one combined with deferasirox) with a return to baseline ALT values.

iii. In 10/40 evaluable patients, four positive for hepatitis C, no increases in ALT were observed. Five of the 10 were prescribed full-dose deferoxamine or deferasirox along with deferiprone; five were receiving deferiprone monotherapy.

Continued deferiprone despite to elevations in ALT. In 11/26 patients in whom ALTs increased, deferiprone was continued. Eight patients on deferiprone monotherapy had elevated ALT for years. Three patients continued on deferiprone, two combined with deferoxamine at 42 mg/kg/day and 16 mg/kg/day, respectively, and one combined with deferasirox 30 mg/kg/day. ALT elevations returned to baseline in three of the eight after 0.5, 0.5, and 5.5 years of deferiprone.

Table 2. Patients with increased ALT (normal <40 U/L) up to January 2016.

Patients [n]	Measurement	Mean±SEM	Median(range)
[26] Increased ALT upon first exposure to DFP [22] or dose escalation [4]; HepC positive [7].	BL ALT	25±4	22(5–75)
	Peak ALT	166±19	139(59–395)
	Fold increase over BL	6.6	6.3(2–24)
	Months between BL and first ALT elevation over BL	3±0.4	2(1–9)
	Months between BL and peak ALT	14.9±2.2	13(1–46)
	FU HIC	15±2	13(1–46)
[21] remained on DFP: [5] ALT normalized; [16] ALT remained >40 U/L.	BL ALT	26±3	24(5–75)
	FU ALT	70±14	55(9–305)
	P value BL to FU	*p < 0.01*	
	Fold increase FU over BL	2.7	2.3(1–19)
	FU HIC	15±3	11(2–43)
[16/21] ALT remains abnormal.	BL ALT	25 ± 4	24(9–69)
	FU ALT	87±17	64(41–305)
	P value BL to FU	*p < 0.003*	
	Fold increase FU over BL	3.5	2.7(1–19)
	HIC at censure of data	18±4	13(2–43)
	Months on deferiprone	49±6	61(13–75)

DFP, deferiprone; HIC, hepatic liver iron concentration; HepC, hepatitis C virus; Baseline, BL; Follow-up, FU; p value indicating worsening ALT, italics, red cell.

https://doi.org/10.1371/journal.pone.0211942.t002

Alteration in regimen. In 15/26 patients in whom ALTs had increased, regimens were altered; in 14/15, ALT elevations resolved completely. In 4/15 patients, deferiprone was withdrawn; ALT returned to pre-deferiprone values within two months. In 3/15, deferiprone dose was reduced (to 70 mg/kg/day, 65 mg/kg/day, and 59 mg/kg/day, respectively); ALT elevations partially (n = 1) or completely (n = 2) resolved over months. In the remaining 8/15 patients, licensed therapy with deferasirox or deferoxamine was added (in parallel, in two patients, with reduction of deferiprone dose); in all 8, ALT returned to pre-deferiprone values within two months.

Re-challenge. Ten of 15 patients were re-challenged with their original regimens: two were re-prescribed deferiprone after it had been withdrawn; two re-prescribed full-dose deferiprone after temporary dose reduction; and six re-prescribed deferiprone monotherapy after an added licensed drug was stopped. In all 10, ALT re-surges followed re-challenge; three are shown in Fig 2.

Overall, after ALT elevations were observed, deferiprone was continued in 21/26 patients. In five, ALT normalized: during monotherapy (3) or after addition of full-dose deferoxamine (1), or deferasirox (1). In 16 patients at the time of writing, ALT remained abnormal, elevated at 87±17 U/L (3.5-fold over baseline) after 49±6 months of exposure ($p < 0.003$).

In deferasirox-treated patients, ALT did not change between baseline (33±5 U/L) and follow-up (36±15 U/L; $p < 0.84$). Seven (12.5%) patients developed elevations over baseline. Four patients continued deferasirox and ALT normalized in three. Three patients resolved on deferoxamine, but rebounded when deferiprone monotherapy was introduced. ALT remained unresolved in two patients but normalized in one patient after 63 months on deferiprone.

New diabetes mellitus during deferiprone. Of 36 non-diabetic patients, 6 (16.6%) were diagnosed with new diabetes during deferiprone (as monotherapy or combined with deferoxamine 7–28 mg/kg/day). In 4/6, HICs closest to the diagnosis of diabetes exceeded 20 mg/g. Of 51 deferasirox-exposed, non-diabetic patients, one (2%), whose parents are diabetic, was diagnosed with diabetes after seven years of excellent compliance with deferasirox; HIC closest to the diagnosis of diabetes was 1.6 mg/g.

GI disturbances. GI disturbances were reported in 7/41 (17%) of deferiprone-treated patients and 6/55 (11%) of deferasirox-treated patients.

Agranulocytosis. Two episodes of agranulocytosis were recorded in patient #3 who 18 years previously developed agranulocytosis requiring hospitalization and infusions of Granulocyte Colony Stimulating Factor (G-CSF) [18] following deferiprone exposure. In 2012, the patient was again prescribed deferiprone monotherapy, despite good ongoing control of iron burden (pre-deferiprone HIC 0.9 mg/g; $T2^*$ 16 msec) during deferasirox therapy. Agranulocytosis again required hospitalization and G-CSF. One month after marrow recovery, deferiprone was prescribed a third time; agranulocytosis again required hospitalization and G-CSF. Repeated exposures to deferiprone after agranulocytosis is contraindicated by published guidelines.

Neutropenia. Neutopenia (Absolute Neutrophil Count <200) developed in another treatment interval, in a different patient, approximately 3.5 years after the introduction of deferiprone monotherapy. No agranulocytosis or neutropenia were reported in deferasirox-treated patients.

Arthralgias. An acknowledged adverse effect associated with deferiprone, arthralgias developed in 11 (27%) patients within months of introduction of deferiprone. This was frequently attributed in the clinical notes to alternative etiologies. In five patients, deferiprone was discontinued: in four patients, 22 to 38 months following onset of arthralgias and, in one patient, after 8 months when HIC was had increased from 23 to 30 mg/g. Arthralgias resolved in all five patients following deferiprone withdrawal. In six patients, deferiprone was not

withdrawn; resolution in five was not recorded, and patient #19 complained of arthralgias until death.

Elevations in serum creatinine. Serum creatinine values exceeding baseline for longer than one month were recorded in 10 (17.8%) deferasirox-exposed patients; in three, the relationship to deferasirox was uncertain. Five patients were switched to deferiprone; in another four, elevations resolved while deferasirox continued; in the tenth, creatinine peaked at 172 μmol/L, and declined to 130 μmol/L while deferasirox continued. No elevations in serum creatinine were reported during deferiprone.

Mechanism of prescription of deferiprone

From 2009 to 2015, deferiprone was not licensed in Canada. There are two possible processes by which an unlicensed drug can be used in patient care in Canada: under the terms a formal research protocol of a (registered) clinical trial [17] or under Health Canada's Special Access Program (SAP) which "considers requests . . .for access to unauthorized drugs for . . .serious or life-threatening conditions when conventional therapies . . . have failed, are unsuitable or unavailable." [22].

Indications for prescription of deferiprone

Deferiprone was prescribed to 41 study patients between 2009 and 2015. We could identify in the EMR no explanation for a proposed switch to deferiprone that was supported by evidence of failure of licensed therapy prescribed as recommended. There was no indication that any patient switched to deferiprone over these six years had "failed" therapy with either deferoxamine or deferasirox. Most patients were recorded as tolerant of at least one and (in most), both licensed first-line chelating agents; some had sustained minor adverse events during deferasirox that had resolved by the time deferiprone was prescribed.

Based upon pre-deferiprone HIC and T2*, the 41 study patients switched to deferiprone could be assigned to one of four groups (**S1 Text. Discussing Indications**). Group I patients (16) had demonstrated optimal responses to licensed therapies; Group II patients (3) had a prolonged absence from chelation related to pregnancy, but licensed therapies had not "failed" and were not "unsuitable or unavailable"; Group III patients (13) had inadequate control of HIC concentration (without evidence of cardiac iron loading) in most following treatment with lower-than-recommended doses of licensed therapy; and Group IV patients (9) had reduced cardiac T2*; half the Group IV patients had, in parallel, the highest HIC recorded in the clinic. According to guidelines, this situation is an indication for therapeutic doses of licensed chelation, not deferiprone.

Discussion

We report the effectiveness and toxicity of the two orally active iron chelator drugs, deferasirox and deferiprone in transfused patients managed at Canada's largest transfusion program. This was not a prospective trial with study protocols, but rather examination of the EMR of patients who consented.

Between 2009 and 2015, one-third of iron-loaded patient were removed from first-line licensed drugs and prescribed regimens involving unlicensed deferiprone. This represents a higher proportion than expected from the literature on patients who have "failed" licensed therapies [3–5]. We were unable to identify in any patient switched to deferiprone evidence of failure, unsuitability, or unavailability of licensed therapies.

During deferiprone monotherapy, body iron burden increased to levels placing patients at risk for glucose intolerance, cardiac disease, and premature death [5, 6]; one death occurred

and new diabetes mellitus, a predictable consequence of uncontrolled iron burden, developed in 17% patients, five times the incidence reported in the modern era of deferoxamine [7]. Body iron burden decreased during deferasirox monotherapy. The frequent elevations in serum ALT observed during exposure to deferiprone in these patients are consistent with our previous observations of hepatic dysfunction arising and hepatic fibrosis progressing during deferiprone therapy, even in patients whose body iron had stabilized [23, 24]. Liver histology was not obtained in the patients in the present study, despite the observed sustained elevations in liver enzymes. Consequently, the etiology and potential hepatic damage associated with the liver enzymes remains undefined [24].

After numerous subsequent studies provided “disparate findings in small series of patients” [15] the FDA approved deferiprone as “last resort” therapy [27], to be prescribed only after all first-line therapies had failed [14], while confirming that no controlled trials had demonstrated a direct treatment benefit [20]. Moreover, the FDA also judged that the year-long study, submitted as ‘pivotal’ evidence for the unique ‘cardio-protective’ effect of deferiprone, [25] did not establish this benefit [26]. Nonetheless, based upon a claimed ‘cardio-protective’ effect [27–29], deferiprone is prescribed worldwide as first line therapy, either as monotherapy (15%), or in regimens combining deferiprone with less-than -therapeutic doses of other drugs (25%) [30, 31]. Deferiprone is frequently prescribed as first line therapy even to children (for example, 68.3% of children in the Middle East [32], despite inadequate monitoring [33–35]). Its recommendation in pediatric practice persists despite higher rates of toxicity in children, including neutropenia (12.6%), agranulocytosis (5%) [35], and liver dysfunction [36, 37]), than were reported in the Apotex-directed “safety” study [34].

The results of this retrospective study of the EMR of patients in one Canadian institution contrast strikingly with the literature. They extend concerns arising from the two prematurely terminated Toronto trials of deferiprone [38], challenging the belief that deferiprone enhances organ-specific iron removal [27–29, 39–41]. In most previous studies of deferiprone, body iron as quantitated by HIC has been unreported, selectively reported, reported after short-term exposures, or reported as unchanged or having worsened [10, 18, 34, 42–74]. By contrast, two Apotex-funded studies have reported declines in HIC during combination therapy (deferiprone combined with deferoxamine) as comparable, or better, than during deferoxamine monotherapy but both studies administered deferoxamine monotherapy at less than 70% therapeutic dose [75, 76]; another uncontrolled study which claimed liver iron declined significantly during combination therapy reported a mean decline (in five patients) which derived from the exceptional reduction of liver iron in one patient.[77].

HIC is the only parameter demonstrated to be directly correlated with body iron [78]. Hence, the lack of HIC data is difficult to reconcile with the idea that body iron is adequately reduced during deferiprone monotherapy or deferiprone when it is combined with less than therapeutic doses of deferoxamine [79].

Rarely discussed with respect to the issue of “combination” therapy is the dose of deferoxamine “combined” with deferiprone. Protocols of deferiprone with low-dose (2–3 infusions/week) deferoxamine, or therapeutic deferoxamine ($\geq$40 mg/kg/day daily) are generally reported under the general rubric of combination therapy, without clarification of dose. For example, deferoxamine doses are reported as “20 to 60 mg/kg/day” [80] or “30 to 45 mg/kg/day, 5 to 7 days per week” [81], which represent between 40% and 120% of recommended dose and therefore cannot be considered a single regimen. No analyses of combination therapy have related the outcomes of therapy to the dose of deferoxamine administered with deferiprone. We were unable to identify a previous publication presenting evidence that deferiprone combined with deferoxamine at a less-than-therapeutic dose ($<$40 mg/kg/day) adequately reduced body iron burden. Our literature review and our present data by contrast indicate that

during combination of deferiprone with deferoxamine, adequate control of body iron is achieved only when full-dose deferoxamine is prescribed. [69, 80–82] This suggests that effectiveness of combination therapy is related to the effectiveness of deferoxamine. Of further concern, combinations of therapy represent off-label prescribing in most jurisdictions, and impose a 3-fold increased risk of adverse events [15].

Despite reports that cardiac iron is 'uniquely' improved by deferiprone [25, 27–29, 39, 41, 79, 83], our data comparing deferiprone and deferasirox showed comparable, and modest, changes in cardiac T2*, with no differences in the proportions of patients estimated as having severe cardiac iron loading. Abnormal T2*s persisted, while HICs remained elevated, or increased in elevation, during years of deferiprone exposure. This is entirely consistent with the acknowledged dynamics of slower cardiac iron deposition and removal compared to those of the liver, [84] and confirms that myocardial iron does not decline in isolation if body iron burden (quantitated by liver iron concentration) is insufficiently treated. Importantly, an industry-sponsored study submitted to FDA as "pivotal" to support superior cardio-protection of deferiprone monotherapy [25] failed to provide evidence for this benefit [26]. By contrast, the (only) independently funded, randomized, double-blinded, placebo-controlled study to have been reported in the literature confirmed a lack of superiority in 'cardio-protection' of deferiprone when combined with full-dose deferoxamine compared to deferoxamine monotherapy [85].

With respect to T2*, most studies of deferiprone monotherapy, often with extended exposures, report no results, no change, improvements in selected patients only, or non-validated endpoints, and cardiac-related deaths [10, 18, 34, 42–61, 63–65, 69, 73, 74, 86–89]. With respect to combination therapy, most studies have not reported cardiac outcomes [57, 60, 61, 63, 64, 75, 90–103]. Uncontrolled studies often combining deferiprone with full-dose deferoxamine have reported changes, [62, 69, 70, 77, 80, 81, 104–109], but of the eight controlled studies in the literature, seven failed to show any difference in cardiac outcomes between combination therapy and deferoxamine monotherapy [65–67, 72, 73, 82, 85]. The eighth study compared a combination of deferiprone and deferoxamine to deferoxamine monotherapy prescribed at lower than therapeutic doses [76].

We observed another outcome not reported in most studies of deferiprone: new diabetes mellitus occurring during deferiprone exposure, as body iron burdens increased or remained elevated. This dreaded complication of poor iron control [5] developed in nearly 17% of deferiprone-exposed patients. One early (Apotex) study reported an incidence of new diabetes in 3.6% deferiprone-exposed patients [56]; however, the glucose intolerance in more than 50% of patients who prematurely stopped deferiprone in that safety study was not reported.

Hepatic dysfunction as reflected by elevations in serum ALT in 65% of UHN patients, sustained over years (**Table 2**), suggests direct hepato-toxicity. Our original concerns about deferiprone-associated liver toxicity [10] were dismissed [27, 28, 39] [110]; the incidence of deferiprone-associated ALT elevations was subsequently estimated as 7.5% [111]. However, most studies in the literature have failed to report ALT changes, or reported lack of changes after short-term exposures, or in selected patients only [10, 18, 44–47, 49, 51, 52, 56, 58, 62, 66–72, 86, 87, 112–114]. One large study recorded ALT surges 3-fold over baseline in 20% of patients [52]; another reported a 4-fold greater mean ALT elevation in deferiprone-exposed patients, than during deferoxamine, but claimed this to be not significantly different [25]. By contrast, the FDA observed that this observation might "signal[s] the potential for deferiprone induced liver toxicity." Often, "transient" ALT elevations [43, 48, 54, 59] have been later acknowledged as sustained: [51, 59, 60] the Apotex "safety" trial, for example, claimed initially that "increased ALT levels "usually stabilized or regressed after three to six months" [34], but

later acknowledged mean ALT had remained significantly elevated over baseline over years [56].

Uniquely, we documented changes in ALT following discontinuation and re-challenge with deferiprone (Fig 2). Because liver enzymes reflect hepatocyte integrity rather than liver function [115], and because we did not record albumin or pro-thrombin values, liver functional status cannot be provided. However, our original concerns about deferiprone and hepatocyte integrity are clearly underscored by these findings.

The irregular monitoring for bone marrow toxicity in one-third of this clinic's patients and the repeated prescribing of deferiprone following episodes of life-threatening agranulocytosis requiring hospitalization violate regulatory agency guidelines, including those of UHN itself [21]. Deaths related to agranulocytosis are reported in under-monitored patients [15] and demand compliance with guidelines specifying with weekly monitoring.

Limitations of this single center analysis include that it does not represent a randomized prospective analysis, a limitation regrettably common in the deferiprone literature. In addition, liver iron concentration and T2* were often not clearly recorded prior to, and following, changes in regimens; as noted, changes were often undertaken without baseline and following data. We circumvented this potential confounder, which would have prevented a clear understanding of the outcomes of effectiveness and safety of different regimens, by defining treatment intervals bracketed by relevant endpoints. Finally, although we have full access to the EMR clinic notes, we are not privy to the reasons why treatment regimens were frequently altered without recording relevant endpoints, or to the rationale behind other clinical decisions not recorded in these notes.

Conclusion

Between 2009 and 2015, one-third of patients transfused and managed in Canada's largest transfusion program were switched from first-line, licensed drugs to regimens of unlicensed deferiprone. Although there is suggestion of a research protocol in this clinic in an abstract [116], it appears that Health Canada's Special Access Program that authorizes use of an unlicensed drug when conventional therapies have failed or are unsuitable or unavailable, was likely used to make deferiprone available to this large proportion of UHN patients. There was no evidence of a failure of first-line therapy in any patient switched to deferiprone.

We provide new evidence of inadequate reduction in hepatic iron, a 17% incidence of new diabetes, and new liver dysfunction in 65% of patients, many who were challenged and re-challenged with deferiprone despite elevated liver enzymes having developed during previous exposure. We identified no evidence of a 'cardio-protective' effect during deferiprone therapy.

Resources to examine these concerns about deferiprone in a prospective controlled study, as urged 20 years ago, will never now be made available. In an era when two highly effective, first-line, chelating agents—one orally active—are available, we caution doctors to reserve deferiprone for patients who have genuinely failed other treatments.

Just as important and of concern are questions raised about the procedures by which a large proportion of patients at the largest Research Institute in Canada were switched from licensed therapies to regimens involving unlicensed deferiprone coincidentally corresponding to market approval of deferiprone. The use of Health Canada's "Special Access Program" and the years of continued exposure of unlicensed deferiprone despite the ineffectiveness and toxicity observed in our study, indicates that issues arising from this analysis are not only scientific. The findings demand an urgent, transparent review of current standards of patient protection, informed consent, and medical practice.

Supporting information

S1 Table. Excluded intervals.
(PDF)

S2 Table. All DFP vs all DFX. All patients exposed to deferiprone compared to all patients exposed to deferasirox: Lab values indicating iron over-load (mean±SEM); Serum ferritin, SF; Hepatic iron concentration, HIC; (#), number of intervals; baseline, BL; follow-up, FU; significant p values, bold.
(PDF)

S3 Table. All patients exposed 30 months. All patients exposed to deferiprone for 31.9±3.7 months compared to all patients exposed to deferasirox at 30±6 months. Lab values of iron over-load (mean±SEM(range)). Serum ferritin, SF; Hepatic iron concentration, HIC; baseline, BL; follow-up, FU; significant p values, bold; p values indicating worsening iron overload, red.
(PDF)

S1 Data. Lab values of included intervals.
(XLSX)

S1 Text. Discussing indications.
(PDF)

Acknowledgments

Christine McClaren and Wen-Pin Chen conducted statistical analyses. Dr. Jacalyn Duffin, Dr. Jocelyn Downie and Dr. Ian Quirt provided editorial review. Students Arshia Javidan and Fred Zhao Xun Feng assisted with figures.

Author Contributions

Conceptualization: Nancy F. Olivieri, Brenda L. Gallie.

Data curation: Nancy F. Olivieri, Amir Sabouhanian, Brenda L. Gallie.

Formal analysis: Nancy F. Olivieri, Amir Sabouhanian, Brenda L. Gallie.

Investigation: Nancy F. Olivieri, Amir Sabouhanian, Brenda L. Gallie.

Methodology: Nancy F. Olivieri, Amir Sabouhanian.

Project administration: Nancy F. Olivieri.

Resources: Nancy F. Olivieri.

Supervision: Nancy F. Olivieri.

Validation: Nancy F. Olivieri, Amir Sabouhanian, Brenda L. Gallie.

Visualization: Amir Sabouhanian, Brenda L. Gallie.

Writing – original draft: Nancy F. Olivieri, Brenda L. Gallie.

Writing – review & editing: Nancy F. Olivieri, Amir Sabouhanian, Brenda L. Gallie.

References

1. Propper RD, Shurin SB, Nathan DG. Reassessment of the use of desferrioxamine B in iron overload. N Engl J Med. 1976; 294(26):1421–3. https://doi.org/10.1056/NEJM197606242942603 PMID: 1272274.

2. Pippard MJ. Iron loading and chelation therapy. In: Weatherall DJ, editor. The thalassemias. Methods in hematology. 6. Edinburgh: Churchill Livingstone; 1983. p. 103–13.
3. Wolfe L, Olivieri N, Sallan D, Colan S, Rose V, Propper R, et al. Prevention of cardiac disease by subcutaneous deferoxamine in patients with thalassemia major. N Engl J Med. 1985; 312(25):1600–3. https://doi.org/10.1056/NEJM198506203122503 PMID: 4000198
4. Zurlo MG, De Stefano P, Borgna-Pignatti C, Di Palma A, Piga A, Melevendi C, et al. Survival and causes of death in thalassaemia major. Lancet. 1989; 2(8653):27–30. PMID: 2567801
5. Brittenham GM, Griffith PM, Nienhuis AW, McLaren CE, Young NS, Tucker EE, et al. Efficacy of deferoxamine in preventing complications of iron overload in patients with thalassemia major. N Engl J Med. 1994; 331(9):567–73. https://doi.org/10.1056/NEJM199409013310902 PMID: 8047080.
6. Olivieri NF, Nathan DG, Macmillan JH, Wayne AS, Liu PP, McGee A, et al. Survival in medically treated patients with homozygous beta-thalassemia. N Eng J Med. 1994; 331(9):574–8. https://doi.org/10.1056/nejm199409013310903 WOS:A1994PD69900003. PMID: 8047081
7. Borgna-Pignatti C, Rugolotto S, De Stefano P, Zhao H, Cappellini M, Del Vecchio G, et al. Survival and complications in patients with thalassemia major treated with transfusion and deferoxamine. Haematologica. 2004; 89(10):1187–93. PMID: 15477202
8. Angelucci E, Brittenham GM, McLaren CE, Ripalti M, Baronciani D, Giardini C, et al. Hepatic iron concentration and total body iron stores in thalassemia major. N Engl J Med. 2000; 343(5):327–31. https://doi.org/10.1056/NEJM200008033430503 PMID: 10922422.
9. Olivieri NF, Brittenham GM. Iron-chelating therapy and the treatment of thalassemia. Blood. 1997; 89(3):739–61. Epub 1997/02/01. PMID: 9028304.
10. Olivieri NF, Brittenham GM, McLaren CE, Templeton DM, Cameron R, McClelland RA, et al. Long-term safety and effectiveness of iron chelation therapy with deferiprone for thalasemia major. N Eng J Med. 1998; 339:417–23.
11. Thompson J, Baird P, Downie J. The Olivieri Report: The complete text of the report of the independent committee of inquiry commissioned by the Canadian Association of University Teachers. Toronto: James Lorimer & Co. Publishers; 2001.
12. Nathan DG, Weatherall DJ. Academic freedom in clinical research. N Engl J Med. 2002; 347(17):1368–71. Epub 2002/10/25. https://doi.org/10.1056/NEJMsb020394 PMID: 12397199.
13. Pazdur R. Complete Response to Application for Ferriprox. OTHER ACTION LETTERS. CENTER FOR DRUG EVALUATION AND RESEARCH APPLICATION NUMBER: 021825Orig1s000. November 30, 2009.
14. Farrell AT. Summary Review of Ferriprox. Application number: 021825Orig1s000. Center for Drug Evaluation and Research. 2011.
15. Fisher SA, Brunskill SJ, Doree C, Chowdhury O, Gooding S, Roberts DJ. Oral deferiprone for iron chelation in people with thalassaemia. Cochrane Database of Systematic Reviews. 2013;(8). https://doi.org/10.1002/14651858.CD004839.pub3 WOS:000323928600028. PMID: 23966105
16. Ward R, Yeo E. Letter to FDA in support of Ferriprox, New Drug Application 021825 http://www.fda.gov/downloads/AdvisoryCommittees/CommitteesMeetingMaterials/Drugs/OncologicDrugsAdvisoryCommittee/UCM271676.pdf2011.
17. Canadian Institutes of Health Research, Natural Sciences and Engineering Research Council of Canada, Social Sciences and Humanities Research Council of Canada. Ethical Conduct for Research Involving Humans 2014. Available from: http://www.pre.ethics.gc.ca/pdf/eng/tcps2-2014/TCPS_2_FINAL_Web.pdf.
18. Olivieri NF, Brittenham GM, Matsui D, Berkovitch M, Blendis LM, Cameron RG, et al. Iron-chelation therapy with oral deferipronein patients with thalassemia major. N Engl J Med. 1995; 332(14):918–22. Epub 1995/04/06. https://doi.org/10.1056/NEJM199504063321404 PMID: 7877649.
19. Brittenham GM. Iron-chelating therapy for transfusional iron overload. N Eng J Med. 2011; 364(2):146–56. https://doi.org/10.1056/NEJMct1004810 WOS:000286142900008. PMID: 21226580
20. Monograph F. Ferriprox Drug Monograph 2011 [cited 2018 6 April]. Available from: https://www.accessdata.fda.gov/drugsatfda_docs/label/2011/021825lbl.pdf.
21. University Health Network Clinical Guidelines 2012. UHN Red Blood Cell Disorders (RBCD) Program2012 6 April 2018:[75–6 pp.]. Available from: https://guides.library.utoronto.ca/c.php?g=250874&p=1672175; https://guides.library.utoronto.ca/ld.php?content_id = 10044946.
22. Canada H. Guidance Document for Industry and Practitioners—Special Access Programme for Drugs. 2008.
23. Olivieri NF, Brittenham GM, McLaren CE, Templeton DM, Cameron RG, McClelland RA, et al. Long-term safety and effectiveness of iron-chelation therapy with deferiprone for thalassemia major. N Engl J Med. 1998; 339(7):417–23. https://doi.org/10.1056/NEJM199808133390701 PMID: 9700174.

24. Brittenham GM, Nathan DG, Olivieri NF, Porter JB, Pippard M, Vichinsky EP, et al. Deferiprone and hepatic fibrosis. Blood. 2003; 101(12):5089–90; author reply 90–1. Epub 2003/06/06. https://doi.org/10.1182/blood-2002-10-3173 PMID: 12788794.

25. Pennell DJ, Berdoukas V, Karagiorga M, Ladis V, Piga A, Aessopos A, et al. Randomized controlled trial of deferiprone or deferoxamine in beta-thalassemia major patients with asymptomatic myocardial siderosis. Blood. 2006; 107(9):3738–44. https://doi.org/10.1182/blood-2005-07-2948 WOS:000237217600061. PMID: 16352815

26. Pazdur R. Complete Response to Application for Ferriprox. OTHER ACTION LETTERS. CENTER FOR DRUG EVALUATION AND RESEARCH APPLICATION NUMBER: 021825Orig1s000. 2009.

27. Neufeld EJ. Oral chelators deferasirox and deferiprone for transfusional iron overload in thalassemia major: new data, new questions. Blood. 2006; 107(9):3436–41. https://doi.org/10.1182/blood-2006-02-002394 PMC1895765. PMID: 16627763

28. Kwiatkowski JL. Real-world use of iron chelators. Hematol-Am Soc Hematol Educ Program. 2011:451–8. https://doi.org/10.1182/asheducation-2011.1.451 WOS:000208759700069. PMID: 22160073

29. Kontoghiorghes CN, Kontoghiorghes GJ. Efficacy and safety of iron-chelation therapy with deferoxamine, deferiprone, and deferasirox for the treatment of iron-loaded patients with non-transfusion-dependent thalassemia syndromes. Drug Design Development and Therapy. 2016; 10:465–81. https://doi.org/10.2147/dddt.s79458 WOS:000369664600001. PMID: 26893541

30. Kwiatkowski JL, Kim HY, Thompson AA, Quinn CT, Mueller BU, Odame I, et al. Chelation use and iron burden in North American and British thalassemia patients: a report from the Thalassemia Longitudinal Cohort. Blood. 2012; 119(12):2746–53. Epub 2012/01/27. https://doi.org/10.1182/blood-2011-04-344507 PMID: 22279056; PubMed Central PMCID: PMCPMC3327453.

31. Ceci A, Mangiarini L, Felisi M, Bartoloni F, Ciancio A, Capra M, et al. The management of iron chelation therapy: preliminary data from a national registry of thalassaemic patients. Anemia. 2011;2011:435683. Epub 2011/07/09. https://doi.org/10.1155/2011/435683 PMID: 21738864; PubMed Central PMCID: PMCPMC3123832.

32. Botzenhardt S, Felisi M, Bonifazi D, Del Vecchio GC, Putti MC, Kattamis A, et al. Long-term safety of deferiprone treatment in children from the Mediterranean region with beta-thalassemia major: the DEEP-3 multi-center observational safety study. Haematologica. 2018; 103(1):e1–e4. Epub 2017/10/29. https://doi.org/10.3324/haematol.2017.176065 PMID: 29079595; PubMed Central PMCID: PMCPMC5777195.

33. Waheed N, Ali S, Butt MA. Comparison of deferiprone and deferrioxamine for the treatment of transfusional iron overload in children with beta thalassemia major. J Ayub Med Coll Abbottabad. 2014; 26 (3):297–300. Epub 2015/02/13. PMID: 25671931.

34. Cohen A, Galanello R, Piga A, Vullo C, Tricta F. A multi-center safety trial of the oral iron chelator deferiprone. In: Cohen AR, editor. Seventh Cooley's Anemia Symposium. 850. New York: The New York Academy of Sciences; 1998. p. 223–6.

35. Botzenhardt S, Sing CW, Wong IC, Chan GC, Wong LY, Felisi M, et al. Safety Profile of Oral Iron Chelator Deferiprone in Chinese Children with Transfusion-Dependent Thalassaemia. Curr Drug Saf. 2016; 11(2):137–44. Epub 2015/10/01. PMID: 26419768.

36. Totadri S, Bansal D, Bhatia P, Attri SV, Trehan A, Marwaha RK. The deferiprone and deferasirox combination is efficacious in iron overloaded patients with beta-thalassemia major: A prospective, single center, open-label study. Pediatr Blood Cancer. 2015; 62(9):1592–6. Epub 2015/03/31. https://doi.org/10.1002/pbc.25533 PMID: 25820920.

37. Songdej D, Sirachainan N, Wongwerawattanakoon P, Sasanakul W, Kadegasem P, Sungkarat W, et al. Combined chelation therapy with daily oral deferiprone and twice-weekly subcutaneous infusion of desferrioxamine in children with beta-thalassemia: 3-year experience. Acta Haematol. 2015; 133 (2):226–36. Epub 2014/11/08. https://doi.org/10.1159/000363210 PMID: 25376266.

38. Olivieri NF, Brittenham GM. Final results of the randomized trial of deferiprone (L1) and deferoxamine (DFO). Blood. 1997; 90(Suppl 1):264a.

39. Hoffbrand AV. Deferiprone therapy for transfusional iron overload. Best Pract Res Clin Haematol. 2005; 18(2):299–317. https://doi.org/10.1016/j.beha.2004.08.026 WOS:000228471500012. PMID: 15737892

40. Berdoukas V, Farmaki K, Wood JC, Coates T. Iron chelation in thalassemia: time to reconsider our comfort zones. Expert review of hematology. 2011; 4(1):17–26. https://doi.org/10.1586/ehm.10.74 WOS:000288146700009. PMID: 21322775

41. Coates TD. Bad liver and a broken heart. Blood. 2014; 123(10):1434–6. https://doi.org/10.1182/blood-2014-01-548552 WOS:000335844600002. PMID: 24627546

42. Agarwal MB, Gupte SS, Viswanathan C, Vasandani D, Ramanathan J, Desai N, et al. Long-term assessment of efficacy and safety of L1, an oral iron chelator, in transfusion-dependent thalassaemia: Indian trial. British Journal Of Haematology. 1992; 82:460–6. PMID: 1419829

43. al-Refaie FN, Wonke B, Hoffbrand AV, Wickens DG, Nortey P, Kontoghiorghes GJ. Efficacy and possible adverse effects of the oral iron chelator 1,2- dimethyl-3-hydroxypyrid-4-one (L1) in thalassemia major. Blood. 1992; 80(3):593–9. PMID: 1638018.

44. Hoffbrand AV, Al-Refaie F, Davis B, Siritanakatkul N, Jackson BF, Cochrane J, et al. Long-term trial of deferiprone in 51 transfusion-dependent iron overloaded patients. Blood. 1998; 91(1):295–300. PMID: 9414297

45. Tondury P, Zimmermann A, Nielsen P, Hirt A. Liver iron and fibrosis during long-term treatment with deferiprone in Swiss thalassaemic patients. British Journal Of Haematology. 1998; 101(3):413–5. PMID: 9633879

46. Mazza P, Anurri B, Lazzari G, Masi C, Palazzo G, Spartera MA, et al. Oral iron chelating therapy. A single center interim report on deferiprone (L1) in thalassemia. Haematologica. 1998; 83:496–501. PMID: 9676021

47. Rombos Y, Tzanetea R, Konstantopoulos K, Simitzis S, Zervas C, Kyriaki P, et al. Chelation therapy in patients with thalassemia using the orally active iron chelator deferiprone (L1). Haematologica. 2000; 85(2):115–7. PMID: 10681716.

48. Lucas GN, Perera BJ, Fonseka EA, De Silva DD, Fernandopulle M. A trial of deferiprone in transfusion-dependent iron overloaded children. Ceylon Med J. 2000; 45(2):71–4. PMID: 11051705.

49. Berdoukas V, Bohane T, Eagle C, Lindeman R, DeSilva K, Tobias V, et al. The Sydney Children's Hospital experience with the oral iron chelator deferiprone (L1). Transfus Sci. 2000; 23(3):239–40. PMID: 11099900.

50. Cohen A, Galanello R, Piga A, Dipalma A, Vullo C, Tricta F. Safety profile of the oral iron chelator deferiprone: a multicentre study. British Journal Of Haematology. 2000; 108(2):305–12. PMID: 10691860

51. Lucas GN, Perera BJ, Fonseka EA, De Silva DD, Fernandopulle M, Karunatilaka DH, et al. Experience with the oral iron chelator deferiprone in transfusion-dependent children. Ceylon Medical Journal. 2002; 47(4):119–21. PMID: 12661341

52. Ceci A, Baiardi P, Felisi M, Cappellini MD, Carnelli V, De Sanctis V, et al. The safety and effectiveness of deferiprone in a large-scale, 3-year study in Italian patients. British Journal Of Haematology. 2002; 118(1):330–6. PMID: 12100170

53. Maggio A, D'Amico G, Morabito A, Capra M, Ciaccio C, Cianciulli P, et al. Deferiprone versus deferoxamine in patients with thalassemia major: a randomized clinical trial. Blood Cells Mol Dis. 2002; 28 (2):196–208. PMID: 12064916

54. Peng CT, Chow KC, Chen JH, Chiang YP, Lin TY, Tsai CH. Safety monitoring of cardiac and hepatic systems in beta-thalassemia patients with chelating treatment in Taiwan. Eur J Haematol. 2003; 70 (6):392–7. PMID: 12756022

55. Galia M, Midiri M, Bartolotta V, Morabito A, Rizzo M, Mangiagli A, et al. Potential myocardial iron content evaluation by magnetic resonance imaging in thalassemia major patients treated with deferoxamine or deferiprone during a randomized multicenter prospective clinical study. Hemoglobin. 2003; 27 (2):63–76. https://doi.org/10.1081/hem-120021538 WOS:000183118000001. PMID: 12779268

56. Cohen AR, Galanello R, Piga A, De Sanctis V, Tricta F. Safety and effectiveness of long-term therapy with the oral iron chelator deferiprone. Blood. 2003; 102(5):1583–7. https://doi.org/10.1182/blood-2002-10-3280 PMID: 12763939

57. Gomber S SR, Madan N. Comparative efficacy of desferrioxamine, deferiprone and in combination on iron chelation in thalassemic children. Ind Pediatr 2004; 41:21–7.

58. Choudhry VP, Pati H, Saxena A, Malaviya A. Deferiprone, efficacy and safety. Ind J Pediatr. 2004; 71 (3):213–6.

59. Taher A, Aoun E, Sharara AI, Mourad F, Gharzuddine W, Koussa S, et al. Five-year trial of deferiprone chelation therapy in thalassaemia major patients. Acta Haematol. 2004; 112(4):179–83. https://doi.org/10.1159/000081268 WOS:000225332800001. PMID: 15564727

60. Peng CT, Wu KH, Wu SF, Liang DC, Yang CP, Jang RC, et al. Deferiprone or deferoxamine vs combination therapy in patients with beta-thalassemia major: A case study in Taiwan. Hemoglobin. 2006; 30 (1):125–30. https://doi.org/10.1080/03630260500455581 WOS:000236010800019. PMID: 16540425

61. Ha SY, Chik KW, Ling SC, Lee ACW, Luk CW, Lam CWK, et al. A randomized controlled study evaluating the safety and efficacy of deferiprone treatment in thalassemia major patients from Hong Kong. Hemoglobin. 2006; 30(2):263–74. https://doi.org/10.1080/03630260600642617 WOS:000237623200016. PMID: 16798652

62. Christoforidis A, Haritandi A, Tsatra I, Tsitourides I, Karyda S, M. A-M. Four-year evaluation of myocardial and liver iron assessed prospectively with serial MRI scans in young patients with b-thalassaemia major: comparison between different chelation regimens. Eur J Haematol. 2006; 78:52–7. https://doi.org/10.1111/j.0902-4441.2006.t01-1-EJH3013.x PMID: 17042760

63. Chen AC, Peng CT, Wu SF, Wu KH, Chiang IP, Tsai CH. Effect of deferiprone on liver iron overload and fibrosis in hepatitis C virus-infected thalassemia. Hemoglobin. 2006; 30(2):209–14. https://doi.org/10.1080/03630260600642518 WOS:000237623200009. PMID: 16798645

64. Aydinok Y, Ulger Z, Nart D, Terzi A, Cetiner N, Ellis G, et al. A randomized controlled 1-year study of daily deferiprone plus twice weekly desferrioxamine compared with daily deferiprone monotherapy in patients with thalassemia major. Haematologica. 2007; 92(12):1599–606. https://doi.org/10.3324/haematol.11414 WOS:000251312100004. PMID: 18055982

65. El-Beshlawy A, Manz C, Naja M, Eltagui M, Tarabishi C, Youssry I, et al. Iron chelation in thalassemia: combined or monotherapy? The Egyptian experience. Ann Hematol. 2008; 87(7):545–50. https://doi.org/10.1007/s00277-008-0471-2 WOS:000256088800004. PMID: 18351337

66. Maggio A, Vitrano A, Capra M, Cuccia L, Gagliardotto F, Filosa A, et al. Improving survival with deferiprone treatment in patients with thalassemia major: A prospective multicenter randomised clinical trial under the auspices of the Italian Society for Thalassemia and Hemoglobinopathies. Blood Cells Molecules and Diseases. 2009; 42(3):247–51. https://doi.org/10.1016/j.bcmd.2009.01.002 WOS:000265810400012. PMID: 19233692

67. Maggio A, Vitrano A, Capra M, Cuccia L, Gagliardotto F, Filosa A, et al. Long-term sequential deferiprone-deferoxamine versus deferiprone alone for thalassaemia major patients: a randomized clinical trial. British Journal Of Haematology. 2009; 145(2):245–54. https://doi.org/10.1111/j.1365-2141.2009.07609.x WOS:000264563600012. PMID: 19236376

68. Won S, Han D, Seo J, al e. Efficacy and safety of deferiprone (Ferriprox), an oral iron-chelating agent, in pediatric patients. Korean J Hematol. 2010; 45:58–61. https://doi.org/10.5045/kjh.2010.45.1.58 PMID: 21120164

69. Kolnagou A, Kleanthous M, Kontoghiorghes GJ. Reduction of body iron stores to normal range levels in thalassaemia by using a deferiprone/deferoxamine combination and their maintenance thereafter by deferiprone monotherapy. Eur J Haematology. 2010; 85(5):430–8. https://doi.org/10.1111/j.1600-0609.2010.01499.x WOS:000283376500007. PMID: 20662901

70. Jamuar SS, Lai AHM, Tan AM, Chan MY, Tan ES, Ng ISL. Use of deferiprone for iron chelation in patients with transfusion-dependent thalassaemia. J Paediatrics and Child Health. 2011; 47(11):812–7. https://doi.org/10.1111/j.1440-1754.2011.02031.x WOS:000297915200012. PMID: 21902752

71. Pepe A, Meloni A, Capra M, Cianciulli P, Prossomariti L, Malaventura C, et al. Deferasirox, deferiprone and desferrioxamine treatment in thalassemia major patients: cardiac iron and function comparison determined by quantitative magnetic resonance imaging. Haematologica. 2011; 96(1):41–7. https://doi.org/10.3324/haematol.2009.019042 WOS:000286902400009. PMID: 20884710

72. Pantalone GR, Maggio A, Vitrano A, Capra M, Cuccia L, Gagliardotto F, et al. Sequential alternating deferiprone and deferoxamine treatment compared to deferiprone monotherapy: Main findings and clinical follow-up of a large multicenter randomized clinical trial in beta-thalassemia major patients. Hemoglobin. 2011; 35(3):206–16. https://doi.org/10.3109/03630269.2011.570674 WOS:000290797500004. PMID: 21599433

73. Cassinerio E, Roghi A, Pedrotti P, Brevi F, Zanaboni L, Graziadei G, et al. Cardiac iron removal and functional cardiac improvement by different iron chelation regimens in thalassemia major patients. Ann Hematol. 2012; 91(9):1443–9. https://doi.org/10.1007/s00277-012-1480-8 WOS:000307286000013. PMID: 22572843

74. Viprakasit V, Nuchprayoon I, Chuansumrit A, Torcharus K, Pongtanakul B, Laothamatas J, et al. Deferiprone monotherapy reduces iron overload in transfusion-dependent thalassemias: 1-year results from a multicenter prospective, single arm, open label, dose escalating phase III pediatric study from Thailand. Am J Hematol. 2013; 88(4):251–60. https://doi.org/10.1002/ajh.23386 WOS:000316808800002. PMID: 23460233

75. Galanello R, Kattamis A, Piga A, Fischer R, Leoni G, Ladis V, et al. A prospective randomized controlled trial on the safety and efficacy of alternating deferoxamine and deferiprone in the treatment of iron overload in patients with thalassemia. Haematologica. 2006; 91(9):1241–3. WOS:000240353100013. PMID: 16956824

76. Tanner MA, Galanello R, Dessi C, Smith GC, Westwood MA, Agus A, et al. A randomized, placebo-controlled, double-blind trial of the effect of combined therapy with deferoxamine and deferiprone on myocardial iron in thalassemia major using cardiovascular magnetic resonance. Circulation. 2007; 115(14):1876–84. https://doi.org/10.1161/CIRCULATIONAHA.106.648790 WOS:000245574900008. PMID: 17372174

77. Tsironi M, Assimakopoulos G, Polonofi K, Rigaki K, Aessopos A. Effects of combined deferiprone and deferoxamine chelation therapy on iron load indices in beta-thalassemia. Hemoglobin. 2008; 32(1–2):29–34. https://doi.org/10.1080/03630260701680474 WOS:000253853400003. PMID: 18274980

78. Angelucci E, Brittenham GM, McLaren CE, Ripalti M, Baronciani D, Giardini C, et al. Hepatic iron concentration and total body iron stores in thalassemia major.[comment][erratum appears in N Engl J Med 2000 Dec 7;343(23):1740]. New England Journal of Medicine. 2000; 343(5):327–31. https://doi.org/10.1056/NEJM200008033430503 PMID: 10922422

79. Kwiatkowski JL. Current recommendations for chelation for transfusion-dependent thalassemia. Tenth Cooley's Anemia Symposium. 1368. New York: The New York Academy of Sciences; 2016. p. 107–14.

80. Farmaki K, Tzoumari I, Pappa C, Chouliaras G, Berdoukas V. Normalisation of total body iron load with very intensive combined chelation reverses cardiac and endocrine complications of thalassaemia major. British Journal Of Haematology. 2010; 148(3):466–75. https://doi.org/10.1111/j.1365-2141.2009.07970.x WOS:000273547000014. PMID: 19912219

81. Berdoukas V, Chouliaras G, Moraitis P, Zannikos K, Berdoussi E, Ladis V. The efficacy of iron chelator regimes in reducing cardiac and hepatic iron in patients with thalassaemia major: a clinical observational study. J Cardiovasc Magn Reson. 2009; 11(1):20. https://doi.org/10.1186/1532-429x-11-20 WOS:000268200000001. PMID: 19558722

82. Kolnagou A, Economides C, Eracleous E, Kontoghiorghes GJ. Long term comparative studies in thalassemia patients treated with deferoxamine or a deferoxamine/deferiprone combination. identification of effective chelation therapy protocols. Hemoglobin. 2008; 32(1–2):41–7. https://doi.org/10.1080/03630260701727085 WOS:000253853400005. PMID: 18274982

83. Hershko C, Cappellini MD, Galanello R, Piga A, Tognoni G, Masera G. Purging iron from the heart. British Journal Of Haematology. 2004; 125(5):545–51. https://doi.org/10.1111/j.1365-2141.2004.04946.x PMID: 15147368

84. Noetzli LJ, Carson SM, Nord AS, Coates TD, JC. W. Longitudinal analysis of heart and liver iron in thalassemia major. Blood. 2008; 112:2973–8. https://doi.org/10.1182/blood-2008-04-148767 PMID: 18650452

85. Porter JB, Wood J, Olivieri N, Vichinsky EP, Taher A, Neufeld E, et al. Treatment of heart failure in adults with thalassemia major: response in patients randomised to deferoxamine with or without deferiprone. J Cardiov Magn Reson. 2013; 15:10. https://doi.org/10.1186/1532-429x-15-38 WOS:000319740100001. PMID: 23688265

86. Del Vecchio GC, Crollo E, Schettini F, Fischer R, De Mattia D. Factors influencing effectiveness of deferiprone in a thalassaemia major clinical setting. Acta Haematol. 2000; 104(2–3):99–102. https://doi.org/10.1159/000039759 PMID: 11154982.

87. Wu SF, Peng CT, Wu KH, Tsai CH. Liver fibrosis and iron levels during long-term deferiprone treatment of thalassemia major patients. Hemoglobin. 2006; 30(2):215–8. https://doi.org/10.1080/03630260600642534 WOS:000237623200010. PMID: 16798646

88. Filosa A, Vitrano A, Rigano P, Calvaruso G, Barone R, Capra M, et al. Long-term treatment with deferiprone enhances left ventricular ejection function when compared to deferoxamine in patients with thalassemia major. Blood Cells Molecules and Diseases. 2013; 51(2):85–8. https://doi.org/10.1016/j.bcmd.2013.04.002 WOS:000320411900004. PMID: 23628348

89. Hejazi S, Safari O, Arjmand R, Qorbani M, Pourrostami K, Safari A, et al. Effect of combined versus monotherapy with deferoxamine and deferiprone in iron overloaded thalassemia patients: a randomized clinical trial. Int J Pediatr. 2016; 4(6):1959–65. WOS:000376611100018.

90. Wonke B, Wright C, Hoffbrand AV. Combined therapy with deferiprone and desferrioxamine. British Journal Of Haematology. 1998; 103(2):361–4. PMID: 9827905

91. Aydinok Y, Nisli G, Kavakli K. Alternate use of deferiprone and desferrioxamine in primary school children with thalassaemia major. British Journal Of Haematology. 1999; 106(1):252–3. PMID: 10444196.

92. Balveer K, Pyar K, Wonke B. Combined oral and parenteral iron chelation in beta thalassaemia major. Med J Malaysia. 2000; 55(4):493–7. PMID: 11221163.

93. Mourad FH, Hoffbrand AV, Sheikh-Taha M, Koussa S, Khoriaty AI, Taher A. Comparison between desferrioxamine and combined therapy with desferrioxamine and deferiprone in iron overloaded thalassaemia patients. British Journal of Haematology. 2003; 121(1):187–9. https://doi.org/10.1046/j.1365-2141.2003.04240.x WOS:000181966200027. PMID: 12670352

94. D'Angelo E, Mirra N, Rocca A, Carnelli V. Combined therapy with desferrioxamine and deferiprone: A new protocol for iron chelation in Thalassemia. Journal of Pediatric Hematology Oncology. 2004; 26(7):451–3. https://doi.org/10.1097/00043426-200407000-00011 WOS:000223113700011.

95. Alymara V, Bourantas D, Chaidos A, Bouranta P, Gouva M, Vassou A, et al. Effectiveness and safety of combined iron-chelation therapy with deferoxamine and deferiprone. Hematology Journal. 2004; 5 (6):475–9. https://doi.org/10.1038/sj.thj.6200550 WOS:000227379500004. PMID: 15570288

96. Kattamis A, Ladis V, Berdousi H, Kelekis NL, Alexopoulou E, Papasotiriou I, et al. Iron chelation treatment with combined therapy with deferiprone and deferioxamine: A 12-month trial. Blood Cells Molecules and Diseases. 2006; 36(1):21–5. https://doi.org/10.1016/j.bcmd.2005.11.002 WOS:000234880000004. PMID: 16386928

97. Ricchi P, Ammirabile M, Spasiano A, Costantini S, Cinque P, Di Matola T, et al. Combined chelation therapy in thalassemia major with deferiprone and desferrioxamine: a retrospective study. Eur J Haematol. 2010; 85(1):36–42. https://doi.org/10.1111/j.1600-0609.2010.01447.x WOS:000278920100006. PMID: 20331740

98. Tamaddoni A, Ramezani MS. Comparison between deferoxamine and combined therapy with deferoxamine and deferiprone in iron overloaded thalassemia patients. Iranian Red Crescent Medical Journal 2010; 12(6):655–9. WOS:000285080500010.

99. Lai ME, Grady RW, Vacquer S, Pepe A, Carta MP, Bina P, et al. Increased survival and reversion of iron-induced cardiac disease in patients with thalassemia major receiving intensive combined chelation therapy as compared to desferoxamine alone. Blood Cells Molecules and Diseases. 2010; 45 (2):136–9. https://doi.org/10.1016/j.bcmd.2010.05.005 WOS:000283380600007. PMID: 20678715

100. Al Hawsawi ZM, Sairafy MH, Tarawah M, Zolaly MA, Rahman A, S AH. Experience with combination therapy of deferiprone and desferrioxamine in β-thalassemia major patients with iron overload at Maternity and Children Hospital, Saudi Arabia. Journal of Taibah University Medical Sciences. 2010; 5 (1):27–35.

101. Mirbehbahani N, Jahazi A, Abad H. The effect of combined therapy with deferoxamine and deferiprone on serum ferritin level of beta-thalassemic patients. Hematology. 2012; 17(3):183–6. https://doi.org/10.1179/102453312X13376952196610 WOS:000305275900010. PMID: 22664119

102. Danjou F, Origa R, Anni F, Saba L, Cossa S, Podda G, et al. Longitudinal analysis of heart and liver iron in thalassemia major patients according to chelation treatment. Blood Cells Molecules and Diseases. 2013; 51(3):142–5. https://doi.org/10.1016/j.bcmd.2013.06.001 WOS:000322942800003. PMID: 23816436

103. Mirbehbahani N, Jahazi A, Amlashi HM, Behnampour N. Comparative efficacy of deferiprone, deferoxamine and combination of deferiprone and deferoxamine on serum ferritin value in beta-thalassemia patients. J Krishna Inst Med Sci Univ. 2015; 4(1):70–6. WOS:000359982400010.

104. Origa R, Bina P, Agus A, Crobu G, Defraia E, Dessi C, et al. Combined therapy with deferiprone and desferrioxamine in thalassemia major. Haematologica. 2005; 90(10):1309–14. WOS:000232614300017. PMID: 16219566

105. Daar S, Pathare A. Combined therapy with desferrioxamine and deferiprone in beta thalassemia major patients with transfusional iron overload. Ann Hematol. 2006; 85(5):315–9. https://doi.org/10.1007/s00277-005-0075-z PMID: 16450126

106. Abdelrazik N. Pattern of iron chelation therapy in Egyptian beta thalassemic patients: Mansoura University Children's Hospital experience. Hematology. 2007; 12(6):577–85. https://doi.org/10.1080/10245330701521614 WOS:000251645500017. PMID: 17852442

107. Perifanis V, Christoforidis A, Vlachaki E, Tsatra I, Spanos G, Athanassiou-Metaxa M. Comparison of effects of different long-term iron-chelation regimens on myocardial and hepatic iron concentrations assessed with T2*magnetic resonance imaging in patients with beta-thalassemia major. International journal of hematology. 2007; 86(5):385–9. https://doi.org/10.1532/IJH97.E0734 WOS:000252493500001. PMID: 18192103

108. Tsiapras D, Fragatou S, Farmaki K, Kyrzopoulos S, Paraskevaidis I, Voudris V, et al. Effect of combined chelation therapy with deferiprone and deferoxamine on left ventricular diastolic function in adult beta-thalassemia major patients. Hemoglobin. 2010; 34(3):210–20. https://doi.org/10.3109/03630269.2010.485120 WOS:000282888200003. PMID: 20524811

109. Alpendurada F, Smith GC, Carpenter JP, Nair SV, Tanner MA, Banya W, et al. Effects of combined deferiprone with deferoxamine on right ventricular function in thalassaemia major. J Cardiovasc Magn Reson. 2012; 14:8. https://doi.org/10.1186/1532-429X-14-8 PMID: 22277065; PubMed Central PMCID: PMCPMC3278357.

110. Wanless IR, Sweeney G, Dhillon AP, Guido M, Piga A, Galanello R, et al. Lack of progressive hepatic fibrosis during long-term therapy with deferiprone in subjects with transfusion-dependent beta-thalassemia. Blood. 2002; 100(5):1566–9. https://doi.org/10.1182/blood-2002-01-0306 PMID: 12176871

111. Saliba AN, El Rassi F, Taher AT. Clinical monitoring and management of complications related to chelation therapy in patients with beta-thalassemia. Expert review of hematology. 2016; 9(2):151–68. https://doi.org/10.1586/17474086.2016.1126176 WOS:000377976500004. PMID: 26613264

112. Hershko C, Hoffbrand AV, Olivieri NF, AlRefaie FN, Tondury P, Wonke B. The International Study Group on Oral Iron Chelators: Results of long-term deferiprone (L1) therapy. British Journal Of Haematology. 1996; 93:570–. WOS:A1996UP11400570.

113. Pootrakul P, Sirankapracha P, Sankote J, Kachintorn U, Maungsub W, Sriphen K, et al. Clinical trial of deferiprone iron chelation therapy in B-thalassaemia/haemoglobin E patients in Thailand. British Journal Of Haematology. 2003; 122(2):305–10. PMID: 12846901

114. Kolnagou A, Kontoghiorghes GJ. Manitenance of normal body range iron store level for up to 4.5 years in Thalassemia Major patients using deferiprone monotherapy. Hemoglobin. 2010; 34(3):204–9. https://doi.org/10.3109/03630269.2010.485890 WOS:000282888200002. PMID: 20524810

115. Giannini EG, Testa R, Savarino V. Liver enzyme alteration: a guide for clinicians. Canadian Medical Association Journal. 2005; 172(3):367–79. https://doi.org/10.1503/cmaj.1040752 WOS:000226492000025. PMID: 15684121

116. Miscevic F, Kuo K, Ward R. Single centre, North American experience with compassionate use of deferiprone in patients with beta-thalassemia major. Blood. 2011; 118(Suppl 1):3185a.
